# The relationship between organisational stressors and mental wellbeing within police officers: a systematic review

**DOI:** 10.1186/s12889-019-7609-0

**Published:** 2019-10-15

**Authors:** Amrit Purba, Evangelia Demou

**Affiliations:** 1 Public Health, Institute of Health and Wellbeing, University of Glasgow, Glasgow, G12 8RZ UK; 2 MRC/CSO Social and Public Health Sciences Unit, Institute of Health and Wellbeing, University of Glasgow, Glasgow, G2 3AX UK

**Keywords:** Police, Officer, Mental health, Wellbeing, Organisational stressor

## Abstract

**Background:**

Occupational stressors in police work increase the risk for officer mental health morbidities. Officers’ poor mental wellbeing is harmful to the individual, can affect professionalism, organisational effectiveness, and public safety. While the impact of operational stressors on officers’ mental wellbeing is well documented, no review has systematically investigated organisational stressor impacts. This study aimed to conduct a systematic review to assess the relationship between organisational stressors and police officer mental wellbeing.

**Methods:**

Systematic review conducted following PRISMA and Cochrane Collaboration guidelines. Literature search was undertaken from 1990 to May 2017 on four databases (EBSCOHOST Medline/SocINDEX/PsycINFO/OVID Embase) and grey literature. Included articles were critically appraised and assessed for risk of bias. Narrative and evidence syntheses were performed by specific mental health outcomes.

**Results:**

In total, 3571 results were returned, and 15 studies met the inclusion criteria. All included studies were published in English between 1995 and 2016, had cross-sectional study designs, spanned across four continents and covered 15,150 officers. Strong evidence of significant associations was identified for organisational stressors and the outcomes of: occupational stress, psychiatric symptoms/psychological distress, emotional exhaustion and personal accomplishment. The organisational stressors most often demonstrating consistently significant associations with mental health outcomes included lack of support, demand, job pressure, administrative/organisational pressure and long working-hours.

**Conclusions:**

This review is the first to systematically examine organisational stressors and mental health in police officers. Organisational stressors that can be targeted by interventions and policy changes to secure officer wellbeing, a healthy work environment, and benefits to the organisation and the public are identified.

## Background

### Mental wellbeing (MW) in police officers

Mental health (MH) disorders are a leading cause of long-term work incapacity and sickness absence [1]. The rise in MH problems over the past decade in the working population has spurred increased public, policy and academic interest [2–4], leading to a focus of research on the role of work environments and lifestyle behaviours on mental wellbeing (MW) across occupational groups [5–7].

The police work environment has many occupational stressors and exposures that can lead to increased risk for mental health morbidities [8]. Policing is one of the most stressful occupations as maintained by academic researchers, police practitioners, health-care professionals and psychologists [9–12] and it ranks in the top three occupations in the Occupational Disease Intelligence Network (ODIN) system for Surveillance of Occupational Stress and Mental Illness (SOSMI) [13]. Police officers experience the same combination of MH issues as the general working population [14, 15]; however, their work is compounded by frequent exposure to inherently dangerous situations, which require a different level of physical and mental ability to respond effectively [16–18]. While this can predispose police officers to stress, some research suggests that individual coping mechanisms can become embedded in police officers personalities, allowing them to cope and accept stressful situations as natural requirements of their role [19, 20] and be more resilient to stress than civilians [5]. Fitness for work is central to professional police standards [21, 22]. Therefore, determining which occupational stressors are related to specific MW outcomes may improve police officer wellbeing and contribute to organisational effectiveness [22].

### Occupational stressors in police work

Intrinsic to police work is the daily experience of intensely stressful situations in often higher frequencies than most other occupations [23, 24]. Constant exposure to people suffering distress and pain, threats to officer safety and wellbeing, having to be in control of emotions when provoked, the inconclusive nature of police work, the responsibility of being in possession of a firearm and more importantly the responsibility of protecting the lives of citizens have been recognised as significant sources of stress [25]. These daily activities are constantly under scrutiny due to the societal and political expectations put on human-service professions [26]. As human-service jobs entail a great deal of interaction with the public, police officers are often expected to display and/or manage particular feelings as part of their job, considered a form of ‘emotional labour’ [27, 28].

Territo and Vetter [29] suggested the stressors affecting police officers could be grouped into four categories (organisational practices and characteristics, criminal justice system practices and characteristics, public practices and characteristics and police work itself). These four categories can be further classified into operational and organisational stressors [30, 31]; the former associated with the very nature of police work, including job-related violence [32], exposure to danger and facing the unknown [32], court overtime [33, 34], and the latter related to organisational administration, management, structure and processes [32].

Organisational stressors have been suggested to be a greater source of stress for police officers [35, 36] because officers may recognise them as oppressive [37], unnecessary [37], unavoidable [37] and uncontrollable [38, 39]. Organisational stressors suggested to contribute to the manifestation of stress include lack of support, heavy work load [32], interpersonal conflict with colleagues and supervisors [40], inadequate resources, time pressure, and an overly bureaucratic organisational system, punitive of staff and strictly managed [40, 41]. These findings seem to hold over cross-cultural comparisons cross the UK [30, 42, 43], USA [44, 45], South Africa [46, 47] and other foreign police agencies [44, 45]. Within the existing evidence base the MW outcomes commonly demonstrating or suggested to be associated with organisational stressors in police officers are occupational stress [48, 49], anxiety [50], depression [50, 51], psychiatric symptoms (PS)/psychological distress (PD) [51, 52], burnout [51, 53] and suicidal ideation [54].

### Rationale

Understanding the risk factors to mental wellbeing in the police workforce is paramount [11, 42, 55], as police play a vital role in the maintenance of society. A previous systematic review [56], assessed coping behaviours adopted by police but did not focus on associations between occupational stressors and MW. The aim of our review, is to examine the associations of organisational stressors in police work with the mental wellbeing outcomes of: occupational stress, anxiety, depression, psychological distress (PD), psychiatric symptoms (PS), burnout (a composite measure of depersonalisation (DP), personal accomplishment (PA) and emotional exhaustion (EE)), and suicidal ideation.

## Methods

### Aim

Systematically review the literature to assess the relationship between organisational stressors and police officer mental wellbeing.

### Review methodology

The review was carried out systematically following the Cochrane Handbook for Systematic Reviews [57] and PRISMA Statement [58] guidance; the narrative synthesis followed the Economic and Social Research Council (ESRC) guidelines [59, 60].

### Eligibility criteria

Inclusion criteria were set priori and were based on the Population, Intervention, Comparison and Outcome (PICO) framework [59, 61] (Additional file 2: Table S2). A scoping search was conducted in EMBASE to pre-test the suitability and adequacy of the PICO criteria. Inclusion criteria included: police personnel from various ranks of any age or gender, including trainees and recruits; studies that considered other occupational groups as well as the police were suitable if a separate analysis of the relationship between organisational stressors and police MW could be extracted (study population); studies identifying one or more organisational stressors in relation to police MW (exposure); MH outcomes measured by general measures of wellbeing such as self-reported perceptions of health status, subjective MH, studies considering physiological, organisational or personal outcomes as well as MW outcomes, were suitable if a separate analysis of the relationship between organisational stressors and police MW outcomes could be extracted (outcomes); all study designs were included.

Studies were excluded if they involved correctional, prison, probation officers, police veterans, military, army and navy personnel, police forensic personnel, civilian (non-sworn police) and traffic police; stressors not inherent in police work; physiological/biological indicators of MH and organisational and personal outcomes (i.e. job satisfaction, job commitment); poster articles and information pieces; and studies that only assessed the prevalence of organisational stressors or MW outcomes.

### Databases and information sources

A comprehensive literature search was conducted from the period of 10th May to 16th May 2017. The electronic medical and social science databases consulted were Medline, PsycINFO, SocINDEX and Embase. In addition, grey literature sources were searched using the same criteria [62]. Prior to checking grey literature sources, a literature search on police accountability and governance was conducted to ensure all professional bodies and regulators relevant to the police force were considered. The final grey literature sources consulted are reported in Additional file 2: Table S3. Additionally, experts in the field of police stress literature were contacted (Personal Communication 1, See Additional file 1: Table S1).

### Search strategy

An initial scoping search with no restrictions or limitations was conducted using a combination of free-text search terms [63]. Returned search results were reviewed to identify potentially relevant subject headings, free-text terms and phrases. The final search strategies were constructed from combinations of MeSH and keywords/free-text terms, adjusted for each database. Search results were limited to studies published in English with no geographic restrictions (potential concerns due to cultural differences were noted [64]). Studies published between 1990 to search date (16th May 2017) were considered. The detailed search strategies are presented in Additional file 2: Tables S4, S5, S6 and S7.

### Study selection

The PICO inclusion criteria were incorporated into an electronic screening tool [65], to standardise the selection process (Additional file 3: Table S8); and piloted on 30 studies [59, 66]. All titles and abstracts were then screened for eligibility using the PICO criteria [67]. Full-texts were screened by one reviewer (AP) and analogous to abstract and title screening, 10% of full-text studies were screened independently by a second reviewer (ED). Hand searching was conducted on the reference lists (AP). Grey literature sources and Google scholar results were also screened (AP) using the electronic screening tool on abstracts and titles. Sources, which could inform the review, but did not satisfy the inclusion criteria were noted. A data extraction form was developed to facilitate and standardise data extraction [59, 68, 69]; this was piloted on 10 included studies [57]. Study authors were contacted if further information or clarification was required (Personal Communication 2, See Additional file 1: Table S1).

### Risk of Bias assessment

Eligible studies were assessed for risk of bias [57, 58], using a previously modified version of the Newcastle-Ottawa Scale (NOS) [70, 71]. Studies scored as high (7–10 stars), intermediate (5–7 stars) or low quality (1–4 stars) [72–75]. The modified NOS, was piloted on five studies prior to use [65]. A single reviewer (AP) assessed and ranked each study based on total score and the results were then discussed with the second reviewer (ED). Data extraction occurred prior to risk of bias assessment, to protect against reporting bias [65].

### Narrative & Evidence Synthesis

The results were narratively synthesised, and findings presented by MW outcome of interest. Evidence synthesis was a stepwise process analysed by MW outcome and was based on study design, methodological quality, consistency with regards to the presence or absence of associations between organisational stressors and specific MW outcomes and the magnitude of these associations [76–78].

The reporting of the organisational stressors was mapped onto the WHO Organisational Stress-related Hazard Categorisation [79]. Six of the nine categories were represented in the findings: *organisational culture*; *workload and work pace; working hours; interpersonal relationships*; *participation and control,* and *career development, status and pay*. For the purposes of this review, workload, work pace and working hours were combined into one category. The evidence synthesis process involved combining the degree of evidence with the magnitude of respective association.

A rating system was used to assess the degree of evidence wherein the modified NOS risk of bias grade of each included study was combined with a second assessment which graded the degree of adjustment by confounders conducted within each included study. The rating system was adapted from previous systematic reviews [71, 76–78] and the underlying developmental process was in accordance with expert recommendations (Personal Communication 3, See Additional file 1: Table S1). Evidence was graded as Strong: *if consistent findings were reported in more than two studies of high quality (at least one study adjusted for participant demographics and additional exposure variables)*; Moderate: *if consistent findings were reported in two studies of high quality or one high quality study and one intermediate quality study, or between more than two studies of intermediate quality (at least one study has adjusted for participant demographics or additional exposure variables)*; and Insufficient: *if identification of only one study or inconsistent findings across studies.*

To assess the magnitude of associations the process included: Step 1-the organisational stressors reported were coded/re-coded under the WHO categories [79] to standardise the reporting of the same/similar organisational stressors presented with different terminology. Step 2 addressed the heterogeneity in the reported measures of effect, including correlation and unstandardized/standardised-beta coefficients, and odds ratios, by developing and applying threshold values previously reported in the literature [71, 76–78] for each effect measure. The final threshold value criteria (see Table 1), were discussed with colleagues having expertise in evidence synthesis (Personal Communication 3, See Additional file 1: Table S1), Using these threshold values (see Table 1), the effect size of each organisational stressor and MW outcome relationship was graded and scored (a weight to indicate each grade’s relative importance) as High (+++/3 points), Intermediate (++/2 points), Low association (+/1 point), No association (−/0 point) and unclear (±/0 point). In Step 3, a weighted average was estimated to account for the levels of relative importance across the five grades by: (i) multiplying the number of effect sizes per grade by the number of points (weight) allocated to that grade; (ii) adding the results across all grades for each MH outcome; and (iii) dividing the total by the sum of the weights (number of grades applied per MW outcome). This weighted average resulted in the mean magnitude of association of included studies by MW outcome. Finally, in Step 4 a RAG (Red, Amber and Green) threshold scale was applied to the overall magnitude of association, where a score of 0 to 1.9 was deemed *‘low/no association’* (Red), 2 to 3.9 was *‘intermediate association’* (yellow), and ≥ 4 was ‘high association’ (green). See Supplementary Material S12 for further detailed process.

**Table 1:** Classification of Measures of Effect

Individual effect measure grades and corresponding score (points)	High (3 points)	Intermediate (2 points)	Low (1 point)	No association (0 points)	Unclear (0 points)
Measures of effect					
Correlation	−1.00 to −0.60/ 0.60 to 1.00	−0.59 to − 0.40/ 0.40–0.59	−0.39 to − 0.01/ 0.01 to 0.39	0	±
Unstandardized or standardised beta coefficient	− 1.50 to − 1.01/ 1.01 to 1.50	−1.00 to − 0.51/ 0.51 to 1.00	−0.50 to − 0.01/ 0.01 to 0.50	0	±
Odds ratio	> 3.00	1.50–3.00	1.01–1.49	1	±

*Note.* Original table compiled using information from Rodriguez-Jareño [76], Bernard [77], Steenstra et al. [78], Krehbiel [80] and Joreskog [81]

## Results

**Fig. 1:**
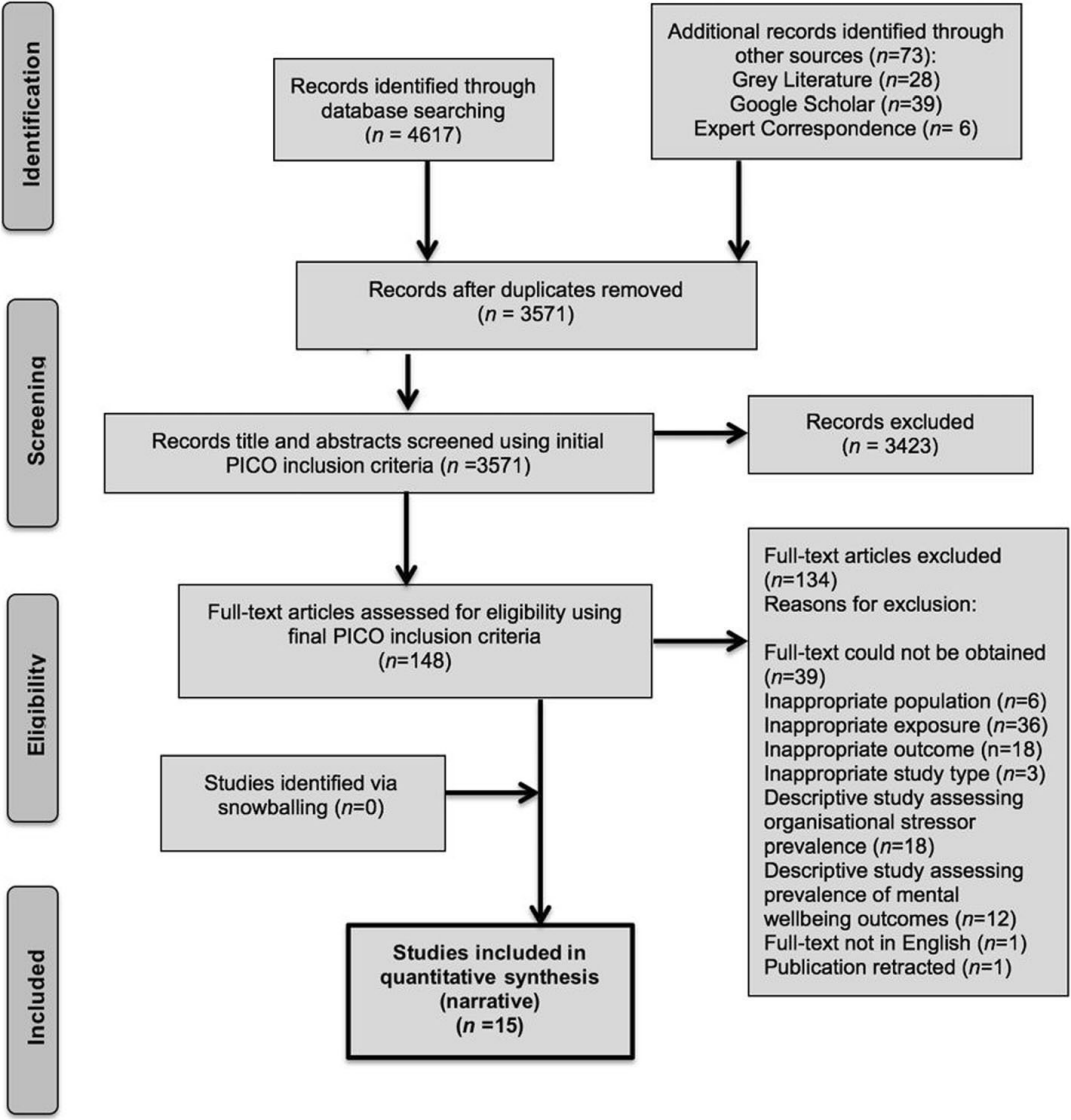
Study selection PRISMA Flow Diagram [58]

The study selection process is documented in Fig. 1. No concerns were raised following second reviewer input in screening titles, abstracts or full-text studies. In total, 15 studies [82–96] met the inclusion criteria and 134 full text studies were excluded (see Fig. 1 and Additional file 4: Table S9).

### Study characteristics

The characteristics of the 15 included studies are summarised in Table 2. All studies were published in English, between 1995 and 2016. No RCTs or cohort studies met the inclusion criteria; all included studies had a cross-sectional study design. The majority were undertaken in the developed world; five from North America [82, 88, 90, 92, 93], five from Europe [84–86, 89, 91], three from Asia [87, 95] and two from Africa [83, 94]. Included studies covered a total of 15,150 male and female police officers, with the smallest study [90] having 78 participants and the largest [86] 3272 participants. Three studies failed to report final response rates [90, 94, 95], the remaining 12 studies included response rates ranging from 33.9% [82] to 96% [96]. The studies which provided information on gender [82–87, 89–94, 96] covered 9706 male and 2592 female study participants; for those that provided mean age, the mean age of participants ranged from 33 to 40 years [82–87, 90, 91, 94]. Two studies [93, 96] adopted an ordinal scale for age; one study [89] dichotomised age; and three studies failed to provide any information on the participant age [88, 92, 95]. Police work tenure ranged from 2.9–17.2 years [82–85, 89, 91, 92, 94, 96]. Only three studies provided information on rank, which included police constables, corporals, sergeants, inspectors, captains, superintendents and senior superintendents [83, 89, 94]. Several studies reported educational background [83, 84, 87, 88, 90–92, 94, 96], marital status [84–86, 90, 91, 94, 96] and race or ethnicity [88, 92–94]. Only one study failed to report participant demographics [95].

**Table 2:** Characteristics of Included Studies

Study ID	Study design	Data collection	Sample size (*n*)	Response rate (%)	Study location	Study population	Study demographics	MW outcome(s) investigated
Adams et al. [82]	Cross-sectional	Self-report online survey	196	33.9	Wisconsin and Illinois, USA	Police officers from 12 police departments in Wisconsin (*n* = 11) or Illinois (*n* = 1)	Male (*n* = 122); female (*n* = 69); did not provide information on gender (*n* = 5) Mean age ± (standard deviation) SD: 39.24 ± 10.33 years Mean tenure ± SD: 11.6 ± 9.01 years	PD EE
Adebayo et al. [83]	Cross-sectional	Self-report questionnaire	214	88.8	Nigeria	Various junior rankings of the Nigeria police	Male (*n* = 132); female (*n* = 82) Mean age ± SD: 33.65 ± 7.28 years Rank: constables (33.3%); corporals (29.0%); sergeants (20.0%); inspectors (16.4%) Education: primary school certificate (14.0%); secondary school certificate (17.8%); national certificate in education / ordinary national diploma (17.8%) Mean tenure ± SD: 11.85 ± 5.37 years	EE
Arial et al. [84]	Cross-sectional	Self-report questionnaire	354	65.9	Switzerland	Police officers from a Swiss cantonal administration. Female officers not included due to small sample size and potential gender effects on symptoms and stressors	Male (*n* = 354) Mean age ± SD: 39.7 ± 8.9 years Education: lower or intermediate educational level (*n* = 341); higher vocational education or university (*n* = 13) Marital status: single, divorced/separated (*n* = 80); married/cohabiting (*n* = 274) Mean tenure ± SD: 14.3 ± 10.1 years; 0–5 years (*n* = 97); > 5–10 years (*n* = 65); > 10–15 years (*n* = 33); > 15–20 years (*n* = 46); > 20–25 years (*n* = 49); more than 25 years (*n* = 64)	PS
Backteman- Erlanson et al. [85]	Cross-sectional	Self-report questionnaire	1554	56.0	Sweden	Police officers who work on patrol	Male (*n* = 419); female (*n* = 437) Mean age ± SD: 37 ± 34 years Marital status: married/living together (*n* = 670) Mean tenure ± SD: 9 ± 11.1 years	EE DP
Berg et al. [86]	Cross-sectional	Self-report questionnaire	3272	51.0	Norway	Police officer members of the largest police industrial organisation in Norway, The Norwegian Police Union (95% of the police service are voluntary members)	Male (*n* = 2692); female (*n* = 501) Mean age ± SD: 38.9 ± 8.7 years; 20–29 years (*n* = 509); 30–39 years (*n* = 1175); 40–49 years (*n* = 1047); 50–59 years (*n* = 430) Marital status: married/common law (*n* = 2715); separated/divorced (*n* = 164)	Anxiety Depression EE DP PA Suicidal Ideation
Chen et al. [87]	Cross-sectional	Self-report questionnaire	832	69.3	Kaohsiung, Taiwan	Police officers in Kaohsiung (population of 1.5 million and about 4300 policemen)	Male (93.3%) (*n* = 776); female (6.7%) (*n* = 56) Mean age ± SD: 39.49 ± 6.65 years; ≤ 34 years (*n* = 202); 35–49 years (*n* = 556); ≥ 50 years (*n* = 74) Education: finished education to junior level (49.9%); senior high school or below (*n* = 264); junior college (*n* = 415); university or above (*n* = 153)	Depression
Crank et al. [88]	Cross-sectional	Self-report questionnaire	1427	71.4	USA	Police chiefs (municipal police organisations) or sheriffs (county police agencies)	Race: Caucasian (*n* = 1279); Hispanic Americans (*n* = 77); African Americans (*n* = 23); Other (i.e. Native American or Korean) (*n* = 25) Education: college education (*n* = 819); post graduate experience (*n* = 221) Police chiefs average years of experience (14 years)	Occupational Stress
Houdmont et al. [89]	Cross-sectional	Self-report online questionnaire	1226	48.0	England	Officers of the federated ranks (constable, sergeant, inspector) from 2 English county forces.	Male (*n* = 721); female (*n* = 505) Age: ≤ 41 years (*n* = 539); ≥ 42 years (*n* = 633) Rank: constable (*n* = 990); sergeant (*n* = 188); inspector (*n* = 48) Departmental tenure: ≤ 9 years (*n* = 1019); 10–19 years (*n* = 184); 20–29 years (*n* = 23) Length of service: ≤ 10 years (*n* = 390); 11–20 years (*n* = 568); 21–30 years (*n* = 268)	PD EE DP PA
Janzen et al. [90]	Cross-sectional	Self-report questionnaire	78	All police employees: 55.4 Response rate for police officers: not reported	Canada	Police officers from a police department of a middle sized Canadian city.	Male (64.0%) (*n* = 50); female (36.0%) (*n* = 28) Mean age ± SD: 36.1 ± 8.0 years Education: university degree or college diploma (68.0%) Marital status: married or living with a partner (82.0%) Policing for 16 years or more (1/3 of respondents) Policing for 6–15 years (24.0%) Policing for 5 years or less (44.0%)	PD
Martinussen et al. [91]	Cross-sectional	Self-report questionnaire	223	45.0	Norway	Norwegian police officers, recruited from one of the larger police districts in Norway	Male (*n* = 173); female (*n* = 48) No gender information (*n* = 2) Mean age ± SD: 36.8 ± 8.30 years Education: mean years of education after primary school± SD: 5.90 ± 1.70 years Marital status: married or living with partner (76.0%) Mean years in current position ± SD: 3.80 ± 3.60 years Employed full time (96.0%)	EE DP PA
McCarty et al. [92]	Cross-sectional	Self-report online survey	2078	57.0	USA	Law enforcement personnel representing 12 law enforcement agencies across the United States	Males (*n* = 1727); females (*n* = 330) Race: African American (*n* = 221); White (*n* = 1299); Hispanic (*n* = 345); Other (*n* = 158) Education: less than BA (*n* = 874); BA or more (*n* = 1106) Mean years on the job ± SD: 17.17 ± 10.15 years	EE
Morash et al. [93]	Cross-sectional	Self-report survey	947	46.2	USA	Police officers from 11 police departments in the USA	Male (*n* = 706); female (*n* = 241) Mean age ± SD: 3.13 ± 0.93 years (ordinal scale ranging from younger than 20 years to older than 60 years) Race: White non- Hispanic (*n* = 778); Other (*n* = 169)	Occupational Stress
Morash et al. [96]	Cross-sectional	Self-report survey	686	96.0	South Korea	Police officers from 11 police departments in Chungbuk Province, South Korea	Males (*n* = 662) Female (*n* = 34) Mean age ± SD: 3.31 ± 1.24 years; > 30 years (88.6%) Education: bachelor’s degree or higher (*n* = 258) Marital status: married or steady state (*n* = 480); no partner (*n* = 206) Mean length of service ± SD: 2.87 ± 1.74 years	Occupational Stress
Mostert et al. [94]	Cross-sectional	Self-report survey	1794	Not reported	South Africa	Police officers from 8 provinces in South Africa	Male (*n* = 1172); female (*n* = 259) Mean age ± SD: 34.53 ± 6.23 years Rank: constable (*n* = 110); sergeant (*n* = 278); inspector (*n* = 775); captain (*n* = 226); superintendent (*n* = 35); senior superintendent (*n* = 7) Race: White (*n* = 574); Black (*n* = 559); Coloured (*n* = 206); Indian (*n* = 56); Race not indicated (*n* = 36) Education: grade 10 (*n* = 140); grade 11 (*n* = 71); grade 12 (*n* = 835); technical college diploma (*n* = 42); tecknikon diploma (*n* = 289); university degree (*n* = 24); postgraduate degree (*n* = 30) Marital status: single (*n* = 283); married (*n* = 787); divorced (*n* = 322); separated (*n* = 26); remarried (*n* = 13) Mean number of years in the police ± SD: 12.98 ± 6.21 years Mean number of years in current position ± SD: 4.28 ± 3.15 years	EE DP
Xavier et al. [95]	Cross-sectional	Self-report survey	296	Not reported	South India	Police officers and sub inspectors from Tamil Nadu	Not reported	Burnout (composite measure of EE, DP and PA) EE DP PA

### Outcomes reported across studies

The included studies reported a number of outcomes of interest including occupational stress [88, 93, 96], anxiety [86], depression [86, 87], PD [82, 89, 90], PS [84], burnout [95] (a composite measure of EE [82, 83, 85, 86, 89, 91, 92, 94, 95], DP [85, 86, 89, 91, 94, 95], PA [86, 89, 91, 95]) and suicidal ideation [86].

### Risk of Bias

All studies were cross-sectional, therefore the modified NOS [70] was used to assess risk of bias. Overall 13 studies were ranked as high [82–91, 93, 94, 96], one as intermediate [92] and one as low quality [95] (Additional file 5: Tables S10, S11). The cross-sectional design of the included studies precludes causal inference [97] and the direction of association cannot be established.

Response rates varied, and all studies employed self-reporting questionnaires. Questionnaires are considered appropriate, as they can provide information about health status, attitudes and behaviours of police officers [98], especially those experiencing MW issues [89]. However, self-reporting questionnaires can lead to method variance bias [99]*,* resulting in overestimation of associations [100] and raise issues of recall bias [101]. Online questionnaires were adopted by three studies [82, 89, 92]. Online surveys often have good data quality and generally confer lower measurement errors, however low response rates are often a challenge [102]. This is demonstrated in these cases, which reported response rates of 33.85 to 57% [82, 89, 92].

Since all studies failed to provide information on the demographics of both respondents and non-respondents, non-response bias cannot be assessed. Response bias may be a problem for topics concerning mental wellbeing, particularly in masculine environments such as policing. One study [88] employed Dillman’s Total Design Method [103] to achieve high response rate and mitigate non-response bias [104]. Eight studies [82, 84, 86, 87, 89, 93, 94, 96] adjusted for additional exposure variables, such as operational stressors and ten studies [83–86, 89–91, 93, 94, 96] controlled for potential confounders, enhancing internal validity.

Eight studies failed to report the sampling method [82, 84, 86, 89, 90, 94–96]. The other studies, adopted probability sampling [83, 85, 88, 91–93] of which one study adopted random gender stratified sampling [85] and four studies used random sampling [83, 88, 91, 92]. One study failed to provide information on the type of probability sample adopted [93]. Purposeful sampling was adopted by one study [87]. None of the studies accounted for officers who were not in work (e.g. court, special assignment, annual leave, off sick) and therefore not included in the study samples. Present state bias cannot be ruled out, if officers more vulnerable to stress have left the profession, creating a more resilient population than that of the general police population [102]. All studies involved voluntary participation, as such all were prone to volunteer bias; which could affect external validity [105].

### Synthesis of results by outcome

All MW outcomes identified in the initial scoping search were reported in the included studies. The results from the included studies are presented by outcome of interest (see also Supplementary material S12–13) [57, 58]. Table 3 shows the organisational stressor and MW outcome associations of included studies by outcome, the assigned effect size grades and corresponding significance levels.

**Table 3:** Organisational Stressor and MW Outcome Associations of Included Studies with Significance level and Effect Size Grade

Mental Wellbeing Outcome(s)	Organisational Stressor(s) and Corresponding Study ID(s)	Grade Assigned to Effect Size	Significance Level	WHO Occupational Stressor Category*
Occupational Stress	Ridicule and set ups [96]	Intermediate	*p* ≤ .01	2
	Bias [93]	Low	*p* ≤ .01	2
	Ridicule and set ups [93]	Low	*p* ≤ .01	2
	Sexual harassment [93]	Low	*p* ≤ .05	2
	Language harassment [93]	Low	*p* ≤ .01	2
	Superiors support [96]	Low	*p* ≤ .01	2
	Lacks influence [93]	Low	*p* ≤ .01	4
	Department issues [88]	Low	*p* ≤ .05	3
	Personnel relations [88]	None	*p* = *ns*	2
	Work group support [93]	None	*p* = *ns*	2
	Sexual harassment [96]	None	*p* = *ns*	2
	Language harassment [96]	None	*p* = *ns*	2
	Lack of advancement opportunity [93]	None	*p* = *ns*	1
Anxiety	Job pressure [86]	Intermediate	*p* ≤ .001	5
	Lack of support [86]	None	*p* = *ns*	2
Depression	Heavy workload [87]	Intermediate	*p* ≤ .004	5
	Judgement from peers [87]	Intermediate	*p* ≤ .004	2
	Job pressure [86]	None	*p* = *ns*	5
	Lack of support [86]	None	*p* = *ns*	2
Psychiatric Symptoms (PS) or Psychological Distress (PD)	Lack of support from superior and organisation [84]	High	*p* ≤ .002	2
	High mental/intellectual demand [84]	Intermediate	*p* ≤ .026	5
	Inadequate work schedule [84]	Intermediate	*p* ≤ .016	5
	≥49 h/week = long working hours [89]	Intermediate	*p* ≤ .05	5
	Internal social stressors [82]	Low	*p* ≤ .01	2
	Effort reward imbalance [90]	Low	*p* ≤ .05	1
	Over-commitment [90]	Low	*p* ≤ .01	1
Burnout	Administrative/organisational pressure [95]	Low	*p* ≤ .01	5
	Police stress [95]	Low	*p* ≤ .01	5
Emotional Exhaustion (EE)	Demand [85]	High	*p* ≤ 0.05	5
	Job pressure [86]	Intermediate	*p ≤* .001	5
	49 h/week = long working hours [89]	Intermediate	*p* ≤ .05	5
	Social support [85]	Intermediate	*p* ≤ 0.05	2
	Lack of support [86]	Intermediate	*p* ≤ .001	2
	Organisational culture [85]	Intermediate	*p* ≤ .05	3
	Organisational climate [85]	Intermediate	*p* ≤ .05	3
	Decision latitude [85]	Intermediate	*p* ≤ .05	4
	Internal social stressors [82]	Low	*p* ≤ .01	2
	Social support from co-workers and supervisors [91]	Low	*p* ≤ .05	2
	Social support [92]	Low	*p* ≤ .05	2
	Perceived workplace fairness [83]	Low	*p* ≤ .01	3
	Unfairness of the organisation [92]	Low	*p* ≤ .05	3
	Lack of resources [94]	Low	*p* ≤ .001	5
	Demand [94]	Low	*p* ≤ .001	5
	Administrative/Organisational pressure [95]	Low	*p* ≤ .01	5
	Police stress [95]	Low	*p* ≤ .01	5
	Overtime work [91]	None	*p* = *ns*	5
	Leadership ([85, 91]	None	*p* = *ns*	3
	Work conflict [85, 91]	None	*p* = *ns*	2
	Autonomy [91]	None	*p* = *ns*	4
Depersonalisation (DP)	Decision latitude [85] Social support [85]	Intermediate Intermediate	*p* ≤ .05 *p* ≤ .05	5 2
	Demand [85]	Intermediate	*p* ≤ .05	5
	Organisational culture [85]	Intermediate	*p* ≤ .05	3
	Organisational climate [85]	Intermediate	*p* ≤ 0.05 ♀; *p* = ns ♂	3
	Social support from co-workers and supervisors [91]	Low	*p* ≤ .001	2
	49 h/week = long working hours [89]	Low	*p* ≤ .05	5
	Demand [94]	Low	*p* ≤ .001	5
	Lack of resources [94]	Low	*p* ≤ .001	5
	Leadership [91]	Low	*p* ≤ .01	3
	Administrative/organisational pressure [95]	Low	*p* ≤ .01	5
	Police stress [95]	Low	*p* ≤ .01	5
	Job pressure [86]	None	*p* = *ns*	5
	Overtime work [91]	None	*p* = *ns*	5
	Work conflict [91]	None	*p* = *ns*	2
	Lack of support [86]	None	*p* = *ns*	2
	Leadership [85]	None	*p* = *ns*	3
	Autonomy [91]	None	*p* = *ns*	4
Personal Accomplishment (PA)	Social support from co-workers and supervisors [91]	Low	*p* ≤ .01	2
	Job pressure [86]	Low	*p* ≤ .001	5
	Administrative/organisational pressure [95]	Low	*p* ≤ .01	5
	Police stress [95]	Low	*p* ≤ .01	5
	Lack of support [86]	None	*p* = *ns*	2
	Work conflict [91]	None	*p* = *ns*	2
	49 h/week = long working hours [89]	None	*p* = *ns*	5
	Overtime work [91]	None	*p* = *ns*	5
	Leadership [91]	None	*p* = *ns*	3
	Autonomy [91]	None	*p* = *ns*	4
Suicidal Ideation	Job pressure [86]	None	*p* = *ns*	5
	Lack of support [86]	None	*p* = *ns*	2

*Note. WHO occupational stressor category: (1) career development, status & pay; (2) Interpersonal relationships; (3) Organisational culture; (4) Participation & control; (5) Workload & working hours

#### Occupational stress

Three high quality studies [88, 93, 96] assessed occupational stress, covering a total of 3060 participants, almost half of which (47%; *n* = 1427) were from one study [88].

Most organisational stressors had low or no association with occupational stress (Table 3). Only ‘ridicule and set-ups’ had an intermediated effect size grade in one study [96], and this was the strongest association observed [88, 93, 96] (Table 3). In this study on South Korean police officers [96], ‘ridicule and set-ups’ was significantly associated with occupational stress (ß = .53, *p* < .01) following adjustment for both participant demographics and additional exposure variables, including sexual/language harassment, feeling invisible, length of service, rank etc. In a study on US officers [93], the same association was graded as low but the relationship remained significant (ß = .12, *p* < .01) following adjustment for participant demographics and other exposure variables, including lack of advancement opportunity or influence, bias, etc.

Dealing with ‘bias’ from co-workers was predictive of occupational stress in US police officers when adjusting for participant demographics and additional exposure variables (ß = .29, *p* < .01) [93]. Officers who exhibited high levels of occupational stress reported stress as a consequence of ethnic or racial bias [93]. Moreover, officers reported considerable time and energy was spent helping co-workers deal with this prejudice and bias, consequently elevating their levels of occupational stress [93]. High levels of ‘superior support’ resulted in low levels of occupational stress in the study conducted on South Korean police officers [96] (ß = -.26, *p* < .01), after adjustment for participant demographics and exposure variables including public disrespect and expressed feelings. However, no evidence of a significant association between high levels of ‘work group support’ and occupational stress was observed (ß = .04, p = *ns*) in US police officers [93]. Τhe organisational stressor poor ‘personnel relations’ (b = .055, p = *ns*) was not significantly associated with occupational stress in a study of police executives either, although should be noted that this study did not account for possible confounding variables [88].

‘Department issues’ (e.g. the department budget, personnel retention and employee labour organisations) was significantly associated (‘low’ effect size grade) with occupational stress (b = .197, *p* < .05) [88]. A ‘lack of influence’ over the way policing is conducted, procedures and policies, was significantly associated with occupational stress (ß = .18, *p* < .01), however ‘lack of advancement opportunity’, (ß = .03, p = *ns*) was not, following adjustment for participant demographics and additional exposure variables [93] .

The organisational stressor ‘language harassment’ was significantly associated with occupational stress in US police officers (ß = .10, *p* < .01) [93], controlling for participant demographics and exposure variables including stigma and appearance and feeling invisible. In the same study, ‘sexual harassment’ was significantly negatively associated with occupational stress (ß = −.08, *p* < .05) [93]. Yet, in the study [96] conducted on South Korean police officers no significant association between occupational stress and either ‘language harassment’ (ß = .07, *p* = *ns*) or ‘sexual harassment’ (ß = .01, *p* = *ns*) was reported.

#### Anxiety

One high quality study, covering 3272 participants, assessed anxiety*,* and demonstrated that ‘job pressure’ was a significant predictor of anxiety symptoms (OR 2.0, 95%; 95%CI:1.5–2.7; *p* < .001) after adjustment for demographics and other exposure variables such as lack of support, subjective health complaints, etc., whereas ‘lack of support’ (OR 1.2; 95%CI: 0.9–1.7; p = *ns*) was not [86].

#### Depression

Two high quality studies [86, 87] covering 4104 participants, with 80% (*n* = 3272) originating from one study [86], assessed depression*.* Significant associations, graded as intermediate (Table 3), were reported between ‘heavy workload’ (OR 1.73; 95%CI:1.19–2.50; *p* = .004) and ‘judgement from peers’ and depression (OR 2.35; 95%CI:1.31–4.23; p = .004) after controlling for exposure variables, such as judgement from peers, little time to spend with families, job performance, etc. [87]. There was no evidence of a significant association between ‘job pressure’ (OR 1.0, 95%CI:0.7–1.4; p = *ns*) or ‘lack of support’ (OR 1.3, 95%CI:0.9–1.99; p = *ns*) and depression in the adjusted models [86].

#### Psychological distress (PD) and psychiatric symptoms (PS)

Four high quality studies assessed psychological distress (PD) and psychiatric symptoms (PS) [82, 84, 89, 90]. These covered 1854 police officers, of which 66% (*n* = 1226) were from one study [89].

The strongest predictor of PS, high effect size grade, was ‘lack of support from superiors and organisation’ (OR 3.58, 95%CI:1.58–8.13; β = 1.28; *p* = .002), after controlling for additional exposure variables such as inadequate work schedule, high mental/intellectual demand, etc. [84]. Intermediate but significant predictors of PS were ‘inadequate work schedule’ (OR 2.84, 95% CI:1.22–6.62; β = 1.04, *p* = .016) and ‘high mental/intellectual demand’ (OR 2.56, 95%CI:1.12–5.86; β = .94, *p* = .026) in adjusted models [84].

For PD, ‘long working hours’ (≥ 49 h/week) demonstrated a high and significant association (OR 2.05, 95%CI:1.57–2.68; *p* < .05), after controlling for age, gender, rank, departmental tenure and years of service [89]; while ‘insider social stressors’- defined as stress arising from co-workers and supervisors- displayed an intermediate but significant association (β = .45, *p* < .01) when controlling for outsider social stressors (e.g. stressor from interactions with civilians/suspects) [82]. The odds of PD caseness in participants working ‘long working hours’, was double that of officers working ‘normal working hours’ (≤48 h/week) following full adjustment [89]. ‘Effort-reward imbalance’ (β = .24, p < .05) and ‘over-commitment’ (β = .40, *p* < .01) were low but significant predictors of PD, after adjusting for age, gender, marital status and education [90].

#### Burnout (defined as a composite measure of EE, DP and PA)

Only one low quality study, assessed burnout as a composite measure of emotional exhaustion (EE), depersonalisation (DP) and personal accomplishment (PA) [95]; but nine studies assessed associations of organisational stressors and EE [82, 83, 85, 86, 89, 91, 92, 94, 95]. Of the latter, six studies further assessed DP [85, 86, 89, 91, 94, 95] and four of these studies additionally assessed PA [86, 89, 91, 95]. Seven of the studies investigating EE, DP and/or PA were of high quality [82, 83, 85, 86, 89, 91, 94], one of medium quality [92], and one of low quality [95]. The studies covered 10,853 participants, 30% of which (*n* = 3272) were from one study [86].

##### Burnout (composite measure of EE, DP and PA)

‘Police stress’ measured by the Police Stress Survey (r = .301, p < .01) and ‘administrative organisational pressure’ (r = .347, p < .01) were significantly correlated and had low associations with burnout, however the authors did not adjust for participant demographics or additional exposure variables [95].

##### Emotional exhaustion (EE)

Four of the nine studies- three of high [85, 86, 94] and one of low [95] quality- investigating EE demonstrated that the strongest predictor of EE was the ‘demand’ inherent in police work. A significant relationship between high ‘demand’ and EE in both male (OR 5.97; 95%CI:3.32–10.71; *p* < .05) and female police officers (OR 7.69; 95%CI:4.21–14.03; p < .05) was reported after adjusting for age [85]. Job ‘demands’ (β = .22, *p* < .001) and ‘lack of resources’ (β = .20, p < .001) both exhibited low but significant relationships with EE, after controlling for both participant demographics and other exposure variables, including conscientiousness, emotional stability, etc. [94]. Intermediate and significant associations were reported for ‘job pressure’ and EE (OR 2.1; 95%CI:1.8–2.5; *p* < .001) [86]. The odds of high EE in police officers working long hours (> 49 h/ week) were double that of officers working normal hours (< 48 h/ week) (OR 1.99, 95%CI:1.52–2.59; *p* < .05) [89]. However, another study found no significant association in adjusted models between ‘overtime work’ and EE (β = .07, p = *ns*) [91].

Similar to burnout, ‘police stress’ (r = .256, *p* < .01) and ‘administrative/organisational pressure’ (r = .310, p < .01) were significantly correlated with EE [95]. Low ‘decision latitude’ demonstrated intermediate and significant associations with EE in both female (OR 2.44; 95%CI:1.38–4.30; *p* < .05) and male police officers (OR 3.94; 95%CI:2.02–7.70; p < .05), following adjustment for age [85]. Yet, lack of ‘autonomy’ was not a significant predictor of EE (β = −.10, p = *ns*) [91].

Several social stressors exhibit intermediate and low associations with EE [82, 85, 86, 91, 92]. Social support from colleagues and superiors is generally associated with lower levels of EE [85, 86, 91, 92], concluded that ‘lack of support’ was significantly associated with EE (OR 1.8; 95%CI:1.5–2.2; p < .001). Martinussen et al. [91] (β = −.25, *p* < .05) and McCarty et al. [92] (b = −.44, p < .05) demonstrated that as levels of social support increased levels of EE decreased; while ‘social support’ was significantly associated with EE in both male (OR 3.47; 95%CI:2.02–5.96; p < .05) and female police (OR 2.79; 95%CI:1.73–4.51; p < .05), after controlling for age [85]. ‘Internal social stressors’ were significantly associated with EE (β = .44, *p* < .01) when controlling for ‘outsider social stressors’ [82]. Work conflict (β = .01, p = *ns*) [91] was not found to be a significant predictor of EE. In the same study, ‘leadership’ was not identified as a significant predictor of EE (β = −.11, p = *ns*) [85, 91]. Similarly, Backteman-Erlanson et al. [85] did not identify a significant association between ‘leadership’ and EE in both male (OR 0.72, 95% CI:0.53–0.99, p = *ns*) and female police officers (OR 0.56, 95% CI:.42–.75, p = *ns*).

In terms of workplace climate and culture, ‘organisational climate’ and ‘organisational culture’ showed intermediate and significant associations with EE in both female (climate: OR 2.48, 95%CI:1.79–3.45; *p* < .05 & culture: OR 2.28, 95%CI:1.61–3.21; p < .05)) and male police (climate: OR 2.17, 95%CI:1.56–3.01; p < .05 and culture: OR 2.09, 95%CI:1.44–3.04; p < .05) following adjustment for age [85]. Two studies, further examined a component of organisational culture by investigating ‘overall perceived fairness’ of police organisations [83, 92]. The first study, reported that 10% of the total variance in EE experienced by participants was attributed to ‘perceived workplace fairness’; and that as ‘perceived workplace fairness’ increased levels of EE decreased (β = −.23, *p* < .01) [83]. Similarly, the second study demonstrated that ‘unfairness of the organisation’ was significantly associated with EE (b = .31, *p* < .05) [92].

##### Depersonalisation (DP)

The organisational stressor demonstrated to be the strongest predictor of DP was low ‘decision latitude’ and this association was identified in both male (OR 2.68; 95%CI:1.37–5.24; p < .05) and female (OR 1.77, 95%CI:1.05–2.99; p < .05) police officers, following adjustment for age [85]. On the contrary, another study did not find any evidence of a significant association between lack of ‘autonomy’ and DP (β = −.04, p = *ns*) after adjustment for confounding [91].

Four studies investigated the impact of high job demand and pressure on DP [85, 86, 94, 95] and three found intermediate [85] and low [94, 95], but significant associations. High job ‘demand’ was significantly associated with DP (β = .11, *p* < .001) after controlling for confounding [94], and similarly was a significant predictor of DP in both male (OR 1.96, 95%CI:1.20–3.20; *p* < .05) and female police officers (OR 2.54, 95%CI:1.57–4.13; p < .05) after adjusting for age [85]. ‘Administrative/organisational pressure’ (r = .218, *p* < .01) and ‘police stress’ (r = .165, *p* < .01) were significantly associated with DP [95]. Conversely, one study found no evidence of an association between ‘job pressure’ and DP (OR 0.9, 95%CI:0.8–1.1; p = *ns*) in the adjusted model [86]. Analogous to EE, ‘long working hours’ (OR 1.30; 95%CI:1.00–1.71; *p* < .05) [89] and ‘lack of resources’ (β = .17, *p* < .001) [94] were significantly associated with DP, while ‘overtime work’ (β = .06, p = *ns*) was not [91] .

Three studies investigated organisational stressors related to interpersonal relationships at work [85, 86, 91]. Similar to EE, high levels of ‘social support’ from co-workers and supervisors resulted in decreased levels of DP (β = −.33, *p* < .001) [91], while the lack of ‘social support’ significantly predicts DP in both male (OR 2.18; 95%CI:1.28–3.71; *p* < .05) and female (OR 1.62, 95%CI:1.06–2.48; p < .05) police officers following adjustment for age [85]. However, one study found no significant association between ‘lack of support’ and DP (OR 0.9, 95% CI:0.8–1.1; p = *ns*) [86] . Similar to EE, there was no significant association between ‘work conflict’ and DP (β = .07, p = *ns*) [91].

Assessing stressors related to organisational culture demonstrated that adoption of appropriate ‘leadership’ significantly decreased DP levels (β = −.24, *p* < .01) [91], but another study found no association between appropriate ‘leadership’ and DP in both male (OR 0.85; 95%CI:0.62–1.15; p = *ns*) and female police officers (OR 0.94, 95%CI:0.73–1.22; p = *ns*) [85]. Poor ‘organisational culture’ was identified as a significant risk factor for DP in both male (OR 1.59, 95%CI:1.12–2.25; p < .05) and female police officers (OR 1.49, 95%CI:1.11–1.99; p < .05), whereas ‘organisational climate’ was as a significant risk factor for female police officers only (OR 1.64, 95%CI: 1.22–2.19; p < .05) and not for males (OR 1.27, 95%CI:0.94–1.73; p = *ns*) [85].

##### Personal accomplishment (PA)

Of the four studies investigating PA, the organisational stressors, social support and job pressure were the strongest predictors of PA, as examined in two high quality studies [86, 91] and one low quality study [95]. ‘Job pressure’ was significantly related to PA (OR 1.3, 95% CI:1.1–1.6; *p* < .001), after controlling for age, gender, lack of support, anxiety, depression, subjective health complaints, suicidal ideation, EE and DP [86]. Similarly, ‘police stress’ (r = .167, *p* < .01) and ‘administrative/organisational pressure’ (r = .152, *p* < .01) were statistically significant correlated with PA, independent of adjustment for confounders [95]. Neither ‘long working hours’ (OR 0.99, 95%CI:0.75–1.32; p = *ns*) [89], nor ‘overtime work’ (β = .01, p = *ns*) [91] were significantly associated with PA.

After adjusting for leadership, work conflict, overtime work, autonomy, work-family pressures, age and gender, high levels of ‘social support’ from co-workers and supervisors (β = .23, p < .01) and PA [91] were significantly correlated. This relationship however was not significant in the study conducted by [86] (OR 1.1, 95% CI: 0.9–1.2; p = *ns*). No significant relationship with PA was observed for ‘leadership’ (β = .13, p = *ns*) [91], ‘work conflict’ (β = −.03, p = *ns*) [91] and ‘autonomy’ (β = .09, p = *ns*) [91] in adjusted models.

#### Suicidal ideation

One high quality study [86] assessed the association between organisational stressors and suicide ideation, covering 3272 participants. This study revealed that neither ‘job pressure’ (OR 0.8, 95%CI:0.6–1.19; p = *ns*) nor ‘lack of support’ (OR 1.3, 95%CI:0.9–1.7; p = *ns*) were significantly associated with suicidal ideation after controlling for confounders [86].

### Evidence synthesis

Thirty-six organisational stressors were identified, of which twenty-five demonstrated significant associations with one or more MW outcomes (Table 3). Overall, a strong degree of evidence with a high magnitude of associations between organisational stressors and MW outcomes (Table 4 and Additional file 6: Table S12 and S13), was observed for the outcomes of PS/PD [82, 84, 89, 90], EE [82, 83, 85, 86, 89, 91, 94, 95] and DP [85, 86, 89, 91, 94, 95]. Strong evidence of intermediate magnitude was identified for studies investigating the relationship between organisational stressors and occupational stress [88, 93, 96] and PA [86, 89, 91, 95]. Studies investigating burnout [95], anxiety [86] and depression [86, 87] provided an insufficient degree of evidence, however the magnitude of associations was rated as intermediate. The degree of evidence and the magnitude of associations were insufficient and low, respectively, for suicidal ideation [86].

**Table 4:** Evidence Synthesis: The MW Outcomes Associated with Organisational Stressors in Police Officers

Mental Wellbeing Outcome(s)	Degree of Evidence	Magnitude of the Association	Study ID(s)
Psychiatric Symptoms (PS) or Psychological Distress (PD)	+++	+++	Adams et al. [82]; Arial et al. [84]; Houdmont et al. [89, 90]
Emotional Exhaustion(EE)	+++	+++	Adams et al. [82]; Adebayo et al. [83]; Backteman-Erlanson et al. [85]; Berg et al. [86]; Houdmont et al. [89]; Martinussen et al. [91]; McCarty et al. [92]; Mostert et al. [94]; Xavier et al. [95]
Depersonalisation (DP)	+++	+++	Backetman- Erlanson et al. [85]; Berg et al. [86]; Houdmont *et el* [89].; Martinussen et al. [91]; Mostert et al. [94]; Xavier et al. [95]
Occupational Stress	+++	++	Crank et al. [88]; Morash et al. [96]; Morash et al. [93]
Anxiety	+	++	Berg et al. [86]
Depression	+	++	Berg et al. [86]; Chen et al. [87]
Burnout	+	++	Xavier et al. [95]
Personal Accomplishment (PA)	+++	++	Berg et al. [86]; Houdmont et al. [89]; Martinussen et al. [91]; Xavier et al. [95]
Suicidal Ideation	+	+	Berg et al. [86]

*Note.* Degree of evidence of included studies by outcome classified as strong, moderate or insufficient. Strong evidence (+++): *Consistent findings in more than 2 studies of high quality. At least one study has adjusted for participant demographics AND additional exposure variables*.; moderate evidence (++): *Consistent findings in 2 studies of high quality or one high quality study and one intermediate quality study, or between more than 2 studies of intermediate quality. At least one study has adjusted for participant demographics OR additional exposure variables*; insufficient evidence *(+): Identification of only one study or inconsistent findings across studies*. Magnitude of association of included studies by outcome based on RAG threshold: high (+++): *≥4*; intermediate (++): *2.0–3.9*; low/no association (+): *0–1.9*

The thirty-six organisational stressors were mapped under the amended WHO Organisational Stress-related Hazard Categories: *organisational culture; workload and working hours; working hours; interpersonal relationships; participation and control and career development.* Within the *organisational culture* category stressors included ‘organisational climate’ and ‘organisational culture’ both of which were significant predictors of EE [85] and DP [85]. ‘Perceived workplace fairness’/‘unfairness of the organisation’ were significant risk factors for EE [83, 92] only. ‘Department issues’ was a significant risk factor for occupational stress [88], while ‘leadership’ was identified as a significant predictor for DP only [91]. ‘Job demands’ [85, 94], high mental/intellectual demand [84], pressure [86, 95] and heavy workload [87], were shown to be significant predictors of PS/PD [84], anxiety [86], burnout [95], EE [85, 86, 94, 95], DP [85, 94, 95], PA [86, 95] and depression [87].

Within the *Workload and working hours* category, ‘long working hours (≥ 49h/week)’ [89] demonstrated an increased risk of PS/PD [89], EE [89] and DP [89] however did not increase risk of PA [89]. A ‘lack of resources’ [94] in the organisation was recognised as increasing the risk of EE [94] and DP [94]. The *Interpersonal relationships* category comprised of stressors including ‘lack of support’, ‘ridicule and set ups’ and ‘bias’. ‘Lack of support’ significantly increased the risk of number of MW outcomes including occupational stress [96], PS/PD [82, 84], EE [82, 85, 91, 92], DP [85, 91], and PA [91]. ‘Ridicule and set ups’ [93, 96], ‘sexual and language harassment’ [93] and ‘bias’ [93] predicted occupational stress, whilst ‘judgement from peers’ was identified as a significant risk factor for depression [87]. There were a small number of stressors which fell under the *Participation and Control* category. Low ‘decision latitude’ was a significant predictor of EE and DP [85], while ‘lacking influence’ in one’s work was predictive of occupational stress [93]. ‘Autonomy’ was not identified as a significant risk factor when investigated in relation to EE, DP and PA [91].

Organisational stressors in the *Career Development* category, included ‘effort-reward imbalance’ [90], ‘over commitment’ [90] and ‘lack of advancement’ [93]. The former two were significant predictors for PD/PS [90], while the latter was not a significant risk factor for occupational stress.

## Discussion

### Summary of findings

This review systematically summarises the organisational stressors intrinsic to police work that significantly contribute to the adverse MW outcomes of occupational stress, anxiety, depression, PS/PD, Burnout, EE, DP, and suicidal ideation. The findings are based on the available evidence established through a systematic search using predefined PICO inclusion criteria set for this review. Fifteen studies met the inclusion criteria, covering relationships between 36 different organisational stressors with MW outcomes, of which twenty-five, including examples such as: organisational culture and climate, perceived workplace fairness/unfairness, leadership, department issues, job demands, high mental/intellectual demand, job pressure, heavy workload, long working hours, lack of resources and support, ridicule and set ups, sexual and language harassment, bias, judgement from peers, low decision latitude, lacking influence, effort-reward imbalance and over commitment, were identified as statistically significant predictors of MW outcomes and demonstrated a strong degree of evidence with high or intermediate magnitudes of associations with the MW outcomes studied..

### Review in the context of previous studies

The majority of the evidence collated for this review has indicated the MW outcomes PS/PD [82, 84, 89, 90], EE [82, 83, 85, 86, 89, 91, 94, 95] and DP [85, 86, 89, 91, 94, 95] demonstrate the strongest relationships with organisational stressors, including lack of support from superiors and organisation, long working hours, inadequate work schedule, high mental/intellectual demand, job demand, organisational climate, organisational culture and low decision latitude. Within the wider literature ‘job demand’ is identified as an important risk factor for the manifestation of MW outcomes, analogous to our review findings. In a systematic review covering the general working population, there was strong evidence that high job demand was a significant predictor of PD [106]. A narrative review of ‘high quality’ longitudinal studies, conducted by de Lange et al., [107] found evidence of causal effects of job demands on PS, concluding that the psychosocial work environment at work is vital for mental health. While this narrative review did not consider police personnel specifically, its purpose was to test the effects of a combination of job characteristics (demand, control and support) on PS [107], all of which are inherent in police work [35].^.^ Our review did not identify any studies investigating the impact of job demand on depression, anxiety and occupational stress, however a review on the general population, demonstrated that high job demands are associated with increased rates of depression, anxiety and occupational stress [1] .Moreover, within the broader law enforcement literature occupational stress and burnout have been reported to arise as a result of high job demands [108]; and with emotional exhaustion in a recent study of German police officers [109], illustrating the spectrum of MW outcomes associated with exposure to this stressor.

On consideration of other organisational stressors related to workload and working hours, job pressure, was identified as the strongest predictor of anxiety, PA and burnout. This finding is in line with reviews on correctional officers where job pressure was identified as a significant predictor of burnout [110, 111] and with a recent study where effort-reward imbalance was positively associated with burnout scores in police officers [112]. Long working hours was identified as increasing the risk of PS/PD, EE and DP in police officers within our review. Two reviews were identified that have investigated the relationship between atypical working hours and MW outcomes in the general working population [113, 114] and the results attest similar findings. One concluded that working more than 48 h a week increased the risk of psychological health difficulties [113], whilst the second concluded that working more than 40 h per week or more than 8 h a day increased the risk of developing symptoms of anxiety or depression [114]. No systematic review or meta-analyses that examined the impact of long working hours on police officers were identified.

The impact of interpersonal relationships was examined within our review. Research on correctional officers have illustrated that relationships with co-workers and the resultant feelings of isolation are significant predictors of occupational stress [110]. Confirming the impact of interpersonal relationships on MW, were the results of a study conducted on 1206 police officers that demonstrated that co-worker discourteous and disrespectful behaviours were significant sources of occupational stress [115]. However, this study did not formally assess associations between stressors and MW outcomes. A recent study showed that job resources (team support, shared values and perceived fairness) predicted wellbeing and decreased EE in police officers [109]. Furthermore, judgement from peers was identified as a significant risk factor for depression, consistent with studies on the general working population which provide strong evidence of relationships between workplace bullying and increased depression symptoms [116]*,* anxiety [117], and stress related psychological symptoms [117]. This emotional demand interpersonal relationships can pose on police officers is often referred to as emotional labour [115], where officers have to manage the display of their emotions and maintain the appropriate demeanour expected by both their work and the greater public. The presence of such interpersonal relationship organisational stressors can have consequential effects, given that police officers rely on colleagues in their work [118].

In the wider organisational stress literature, high levels of social support at work from colleagues and supervisors have been found to be protective of mental health [5, 106, 110, 119]. Systematic reviews on the general working population have indicated that low levels of support result in increased levels of PD [106] and predict the onset of depression [119]. Another review of 14 longitudinal studies revealed that lack of social support enhanced depression [120], and a review on correctional officers demonstrated increased levels of occupational stress resulting from lack of support [110]. A study conducted on a special police force unit demonstrated lack of support was a significant risk factor for DP [5]. Similarly, our review identified that low levels of social support resulted in an increased risk of a number of MW outcomes including occupational stress [96], PS/PD [84], EE [86, 91, 92], DP [85, 91] and PA [91]. Only one study in our review investigated the relationship with anxiety and found no evidence of an association [86], whereas no study investigated the relationship between social support and depression.

Lack of support showed no association with suicidal ideation, although only one study investigated this relationship [86]. Only two systematic reviews exist, to the best of our knowledge, which investigate the issue of suicide in the police – Cantor et al. [121] and Hem et al. [122]. Following a review of ten studies, four of which had sample size of 10 or less, Cantor et al. [121] reported evidence of elevated suicide rates in police officers, however specific relationships between suicide and organisational stressors could not be extracted in this study. Hem et al. [122] compared levels of suicide in the police with the general working population and contrarily reported no elevated suicide rates in police officers.

### Strengths & Limitations

The primary strength of this review is that it is the first to our knowledge that examines associations across a number of organisational stressors and police officer MW outcomes. Included studies either only looked at police officer populations or carried out sub-analyses, that allowed the relationship between organisational stressors and police officer MW to be extracted. The review was performed and reported in accordance with guidance for undertaking a systematic review [57, 63], adhered to the PRISMA checklist [58], and adopted guidelines for the narrative synthesis where possible [60], making it methodologically robust and reproducible. While the study was not included in an international review database, we have documented every step in a transparent and reproducible fashion (see Additional files 1, 2, 3, 4, 5 and 6). One reviewer undertook selection/assessment of studies, with a proportion checked by second reviewer, to reduce bias and enable discrepancies to be resolved via discussion [57]. Expert opinions and advice were sought from systematic review and epidemiology academics on the development of the research protocol.

All included studies were rated as either high (13 of the 15 included studies) or intermediate (1 of the 15 included studies) quality and adopted self-reporting validated measures for both exposure and outcome data.

Summarising the evidence without or with incomplete statistical pooling has been advocated as useful for reviews but can be considered arbitrary and subjective [123, 124]. Whilst, the labels adopted within the evidence synthesis, should be interpreted with caution, the advantage of the followed strategy is that the underlying process is explicit and reproducible.

The studies included in this review were undertaken across four continents, i.e. Europe, North America, Africa and Asia. In general, all demonstrated similar findings regarding the associations between specific organisational stressors and MW outcomes in police officers, with some differences noted, thereby strengthening the generalizability of the results on an international scale.

As with all systematic reviews, new potentially eligible studies may have been published since the literature search was conducted, which could be a limitation. We have identified three studies that have been published since our search that examine organisational risk factors (shift work [36], job demands and resources [109] and supervisor support [125]) and mental health outcomes (stress [36, 125], EE and wellbeing [109]). The results of these studies are in agreement with the outcomes of our review [109, 125], and present a new significant association between shift work and occupational stress [36]. Additionally, a number of potentially eligible studies could not be accessed in full-text. In addressing this limitation, efforts were made to document the studies which could not be accessed, as well as the efforts undertaken to attempt to retrieve these studies (see Additional file 7: Table S14). Whilst strategies were employed to reduce the odds of missing studies on this subject, the chance that a study was omitted cannot be excluded. Moreover, due to the cross- sectional nature of all included studies, causal relationships could not be established. As demonstrated the significant relationships are complex and of varying strengths, with many stressors occurring concurrently and impacting on numerous outcomes. It cannot be discounted that some organisational stressor and MW outcome relationships have not been identified in the evidence collated for this review. Moreover, due to the paucity of literature on this topic it is possible that a number of organisational stressor and MW outcome relationships still require investigation.

### Public health and policy implications

Beneficiaries from a mentally healthy police workforce include the police officers themselves, police organisations, their families, and the public [126]. Reducing poor police officer MW can increase morale, productivity, effectiveness, efficiency and general wellbeing [127], as well as having the potential to reduce compensations claims, on-the-job accidents, civil liabilities for counter-productive behaviour, early retirement and negative perceptions from both the media and public [127].

This review has highlighted the organisational stressors which can be targeted by policies and interventions to reduce the hazard they pose to police officer MW. The organisational stressors shown to impact on police officer mental wellbeing, including lack of support, demand, and interpersonal relationships with colleagues and supervisors, are all amenable to change. It is important therefore to identify the interventions, workplace and other policy changes which address these organisational stressors to promote optimal MW in police officers and these should be incorporated into policing organisational and public health strategies [82]. In addressing lack of support, potential interventions could be aimed at changing the police culture by expanding training and promotion programmes. Flattening the hierarchal structure has also been proposed [41]. Moreover, training specifically for police leaders has been recommended, focusing on awareness of the organisational stressors their employees face, to help reduce their occurrence and mitigate their effects [88].

The success of the recommendations outlined, rely on the resources available to the policing profession. Budget cuts, for instance in the UK police force, in the past decade have seen a decrease in police officer numbers, therefore increasing the demand placed on active officers [128]. Scarce resources allocation could be optimised through increasing police officer numbers; prioritising interventions aimed at promoting support seeking and support services for police officers, therefore shifting the police culture from one that values self-reliance and stoicism to one that promotes the overall wellbeing of their employees [129].

## Conclusion

The findings of this review, examining the relationship between organisational stressors and MW in police officers, provide evidence of an association between organisational stressors and occupational stress, depression, PS/PD, Burnout, EE, DP, PA. Those organisational stressors which demonstrated significant relationships with the MW outcomes considered included lack of support from colleagues, supervisors and the organisation, ridicule and set ups, job demand and pressure, and long working hours.

The evidence identified suggests that due to the extent to which police organisational culture, structure and practice can create stressors, strategies which address how officers treat each other, promote support seeking for mental health issues, and provide police leaders with the knowledge to identify and mitigate occupational stress, could be the most effective. However, there is still a lack of evidence surrounding many organisational stressors and specific MW outcomes and especially a lack of evidence on the effectiveness of proactive and reactive strategies to reduce occupational stressors within policing, reinforcing the need for further research. The evidence base should be enhanced with more longitudinal studies, including understudied factors such as interpersonal conflicts and emotional demands [106]. Advancement in this field can lead to improvement in the MW of this occupational group and concomitantly result in benefits for both policing organisations as well as the greater public in which they serve.

## Supplementary information

**Additional file 1: Table S1.** Personal Communications with experts.

**Additional file 2: Tables S2, S3, S4, S5, S6** and ** S7.** PICO Statement (S2), Grey literature sources (S3), and Search Strategies Adopted for Systematic Review (Table S4-S7).

**Additional file 3: Table S8.** PICO Screening Template (Table S8).

**Additional file 4: Table S9.** Excluded Studies with Reason for Exclusion (Table S9).

**Additional file 5: Tables S10** and **S11.** Results of Modified NOS Risk of Bias Assessment for all Included Studies (Table S10) and Summary of Included Studies (Table S11).

**Additional file 6: Tables S12** and **S13.** Process Adopted to Determine Overall Magnitude of Association of Included Studies by Outcome (Table S12) and Overall Degree of Evidence Grade by MW Outcome (Table S13).

**Additional file 7: Table S14.** Eligible Studies following Title and Abstract Screening, which could not be accessed in Full-text (Table S14).

## Data Availability

All data collected from the literature and analysed during this study are included in this published article and its supplementary information files.

## Abbreviations

CRD: Centre for Reviews and Dissemination
DP: Depersonalisation
DRPST: Disaster Related Psychological Screening Test
ESRC: Economic and Social Research Council
GHQ: General Health Questionnaire
HADS: Hospital Anxiety and Depression Scale
HMICS: Her Majesties Inspectorate of Constabulary Scotland
HSE: Health and Safety Executive
ICMJE: International Committee of Medical Journal Editors
JSS: Job Stress Survey
MBI: Maslach Burnout Inventory
MBI-GS: Maslach Burnout Inventory- General Survey
MBI-HSS: Maslach Burnout Inventory-Human Service Survey
MH: Mental Health
MW: Mental Wellbeing
NOS: Newcastle Ottawa Scale
ODIN: Occupational Disease Intelligence Network
ONS: Office for National Statistics
PA: Personal Accomplishment
PD: Psychological Distress
PS: Psychiatric Symptoms
PSS: Police Stress Survey
RAG: Red, Amber, Green
SOSMI: Surveillance of Occupational Stress and Mental Illness
WHO: World Health Organisation
