# Supplementary material for: The relationship between organisational stressors and mental wellbeing within police officers: a systematic review
Source: BMC Public Health. 2019 Oct 15;19:1286. doi: 10.1186/s12889-019-7609-0 (PMC6792329; doi:10.1186/s12889-019-7609-0)
Supplement: Supplementary file 1 — Additional file 1: Table S1. Personal Communications with experts. [file 12889_2019_7609_MOESM1_ESM.docx]

The Relationship between Organisational Stressors and Mental Wellbeing within Police Officers: A Systematic Review

Additional File 1

File Format: DOC

Title: Table S1

Description: Personal Communications with experts

Table S1

Personal Communications with experts

| Personal Communication 1 | Morash. M, email communication, June 12, 2017  Violanti. J, email communication, May 22, 2017  Brown. J, email communication, June 22, 2017 |
| --- | --- |
|  |  |
| Personal Communication 2 | Morash.M, email communication, June 12, 2017  Xavier. P, email communication, June 12, 2017  Backteman-Erlanson. S, email communication, June 12, 2017  Martinussen.M, email communication, June 12, 2017  McCarty. W, email communication, June 12, 2017 |
|  |  |
| Personal Communication 3 | Johnman. C, personal communication, June 29, 2017  Campbell. M, personal communication, June 15, 2017 |
