# Supplementary material for: The relationship between organisational stressors and mental wellbeing within police officers: a systematic review
Source: BMC Public Health. 2019 Oct 15;19:1286. doi: 10.1186/s12889-019-7609-0 (PMC6792329; doi:10.1186/s12889-019-7609-0)
Supplement: Supplementary file 2 — Additional file 2: Tables S2, S3, S4, S5, S6 and S7. PICO Statement (S2), Grey literature sources (S3), and Search Strategies Adopted for Systematic Review (Table S4-S7). [file 12889_2019_7609_MOESM2_ESM.docx]

The Relationship between Organisational Stressors and Mental Wellbeing within Police Officers: A Systematic Review

Additional File 2

File Format: DOC

Title: Table S2-S7

Description: PICO Statement (Table S2); Grey literature sources (Table S3); Search Strategies Adopted for Systematic Review (Table S4-S7)

Table S2

PICO Statement

| PICO Component | Criteria |
| --- | --- |
| Population | - Police personnel from various ranks within the police hierarchy of any age or gender, including trainees and recruits. - Studies that considered other occupational groups as well as the police were deemed suitable for inclusion if a separate analysis of the relationship between organisational stress and police MW could be extracted.   *Note.* For the purpose of this review police personnel were defined as warranted law employees of a police force(1).The decision to use the term ‘police personnel’ was made to ensure optimal retrieval of relevant articles due to the varying use of the term ‘police officer’ on an international scale. |
| Exposure (Intervention) | - Studies identifying one or more organisational stressors in relation to police MW. - Stressors inherent in police work (i.e. work load, job demand, reward, opportunities, work pressure). - Stressors arising internally from police department practices and policies (i.e. leadership, supervision, support, communication, organisational culture, climate, structure and hierarchy). - External stressors stemming from the criminal justice system (i.e. organisational justice). - Internal stressors confronting individual officers (i.e. corruption, incivility, bullying and violence).   *Note.* Studies that considered operational stressors in conjunction with the organisational stressors listed above were deemed suitable for inclusion if a separate analysis of the relationship between the organisational stressors and police MW could be extracted. The Intervention component was considered as Exposure within this review. |
| Outcome | - Mental health outcomes measured by general measures of wellbeing such as self-reported perceptions of health status (i.e. occupational stress, depression, anxiety or burnout). Studies may include subjective mental health outcomes (i.e. helplessness, isolation, meaninglessness, insecurity, quality of life, quality of work life, morale, disengagement, powerlessness and detachment). - Studies considering physiological, organisational or personal outcomes as well as MW outcomes, were deemed suitable for inclusion if a separate analysis of the relationship between organisational stressors and police MW outcomes could be extracted. |
| Study Design | - Published and non-published studies (accessed through contact with key authors), peer reviewed literature, doctoral dissertations, masters theses and conference papers/reports were considered.   *Note.* The decision to include all study designs was based on the limited number of relevant studies identified following the initial scoping search. Therefore, no exclusions were made with regards to the study design of included studies. |

Table S3

Grey Literature Sources Consulted

| Grey Literature Sources Consulted |
| --- |
| Association of Police and Crime Commissioners (APCC)  Audit Scotland  British Transport Police Authority (BTPA)  Google Scholar  Health and Safety Executive (HSE)  Her Majesty’s Inspectorate of Constabulary (HMIC)  Home Office  Independent Police Complaints Commission (IPCC)  Metropolitan Police Federation (MPF)  National Technical Reports Library US (NTIS)  Opengrey.eu  Police Federation of England and Wales (PFEW)  Scottish Police Authority (SPA)  UNISON |

Table S4

Medline Search Strategy

Interface: EBSCOhost

Search date: 16/05/2017

Search screen: Advanced Search; Search modes: Boolean/Phrase

Medical subject heading (MeSH) term: MH; Title: TI; Abstract: AB

| No. | PICO | Search Term | Limits applied | Hits |
| --- | --- | --- | --- | --- |
| S1 | Population | (MH "Police") OR (MH "Law Enforcement") |  | 6,800 |
| S2 |  | (TI police* OR AB police*) OR (TI “law enforce*” OR AB “law enforce*”) OR ( TI "blue coat*" OR AB "blue coat*" ) OR (TI garda* OR AB garda*) OR (TI SWAT OR AB SWAT) |  | 15,869 |
| S3 |  | S1 OR S2 |  | 19,033 |
| S4 | Organisational Structure | (TI "organi?ational structure*" OR AB "organi?ational structure*”) OR (TI "workplace structure*" OR AB "workplace structure*") OR (TI "work structure*" OR AB "work structure*") OR (TI "police structure*" OR AB "police structure*") OR (TI "rank structure*" OR AB "rank structure*") OR (TI "organi?ational hierarch*" OR AB "organi?ational hierarch*”) OR (TI "workplace hierarch*" OR AB "workplace hierarch*”) OR (TI "work hierarch*" OR AB "work hierarch*”) OR (TI "police hierarch*" OR AB "police hierarch*”) |  | 3,219 |
| S5 | Organisational Stressors | (MH "Workload") OR (MH "Organizational Culture") OR (MH "Reward") OR (MH "Salaries and Fringe Benefits") OR (MH "Conflict (Psychology)") OR (MH "Workplace Violence") OR (MH "Professional Misconduct") OR (MH "Bullying") OR (MH "Sexual Harassment") OR (MH "Harassment, Non-Sexual") OR (MH "Interprofessional Relations") OR (MH "Leadership") OR (MH "Personnel Turnover") OR (MH "Personnel Downsizing") OR (MH "Resource Allocation") OR (MH "Absenteeism") |  | 173,302 |
| S6 |  | (TI "organi?ational demand*" OR AB "organi?ational demand*”) OR (TI "workplace demand*" OR AB "workplace demand*”) OR (TI "work demand*" OR AB "work demand*”) OR (TI "job demand*" OR AB "job demand*”) OR (TI "work load*" OR AB "work load*”) OR (TI "workload*" OR AB "workload*”) OR (TI "heavy caseload*" OR AB "heavy caseload*”) OR (TI "work activit*" OR AB "work activit*") OR (TI "working hour*" OR AB "working hour*") OR (TI overtime OR AB overtime) OR (TI "job strain" OR AB "job strain”) OR (TI "work related strain" OR AB "work related strain”) OR (TI "work control*" OR AB "work control*”) OR (TI "workplace control*" OR AB "workplace control*”) OR (TI "job control*" OR AB "job control*”) OR (TI "decision latitude" OR AB "decision latitude") OR (TI "work pressure*" OR AB "work pressure*") OR (TI "job pressure*" OR AB "job pressure*") OR (TI "workplace pressure*" OR AB "workplace pressure*") OR (TI "time pressure*" OR AB "time pressure*") OR (TI “working condition*” OR AB “working condition*”) OR (TI "physical danger*" OR AB "physical danger*") OR (TI “work environment*” OR AB “work environment*”) OR (TI "organi?ational environment*" OR AB "organi?ational environment*") OR (TI "organi?ational climate*" OR AB "organi?ational climate*") OR (TI "work climate*" OR AB "work climate*") OR (TI "organi?ational culture*" OR "organi?ational culture*") OR (TI "work culture*" OR AB "work culture*") OR (TI "police culture*" OR AB "police culture*") OR (TI "demand and control" OR AB "demand and control") OR (TI "demand control support" OR AB "demand control support") OR (TI "skill utili?ation" OR AB "skill utili?ation") OR (TI "job skill*" OR AB "job skill*”) OR (TI "professional worth*" OR AB "professional worth*") OR (TI "organi?ational communication*" OR AB "organi?ational communication*") OR (TI "role responsib*" OR AB "role responsib*") OR (TI "police responsib*" OR AB "police responsib*") OR (TI "police power*" OR AB "police power*") OR (TI "role requirement*" OR AB "role requirement*") OR (TI "role ambiguity" OR AB "role ambiguity") OR (TI "role problem*" OR AB "role problem*") OR (TI reward* OR AB reward*) OR (TI salar* OR AB salar*) OR (TI pay OR AB pay) OR (TI wage* OR AB wage*) OR (TI " opportunit*" OR "opportunit*") OR (TI "career develop*" OR AB "career develop*") OR (TI "promotion prospect*" OR AB "promotion prospect*") OR (TI "career prospect*" OR AB "career prospect*") OR (TI “career advance*” OR AB “career advance*”) OR (TI “employment insecurit*” OR AB “employment insecurit*”) OR (TI “employment securit*” OR “employment securit*” ) OR (TI “job security” OR AB “job security”) OR (TI “job insecurit*” OR AB “job insecurit*”) OR (TI conflict* OR AB conflict*) OR (TI violence OR AB violence) OR (TI incivil* OR AB incivil*) OR (TI uncivil OR AB uncivil) OR (TI corrupt* OR AB corrupt*) OR (TI aggress* OR AB aggress*) OR(TI misconduct OR AB misconduct) OR(TI brutality OR AB brutality) OR(TI racism OR AB racism) OR (TI unrest OR AB unrest) OR(TI cynic* OR AB cynic*) OR (TI hostil* OR AB hostil*) OR (TI bullying OR AB bullying) OR (TI harass* OR AB harass*) OR (TI discontent* OR AB discontent*) OR (TI “blame culture*” OR AB “blame culture*”) OR (TI “interprofessional relation*” OR AB “interprofessional relation*”) OR (TI “police manage*” OR AB “police manage*”) OR (TI leader* OR AB leader*) OR (TI “supervisory influence*” OR AB “supervisory influence*”) OR (TI support* OR AB support*) OR (TI “police supervision” OR AB “police supervision”) OR (TI “police administration*” OR AB “police administration*”) OR (TI consultation* OR AB consultation*) OR (TI “occupational education” OR AB “occupational education”) OR (TI “occupational training” OR AB “occupational training”) OR (TI “organi?ational justice” OR AB “organi?ational justice”) OR (TI “organi?ational injustice*” OR AB “organi?ational injustice*”) OR (TI “criminal justice*” OR AB “criminal justice*”) OR (TI “personnel turnover” OR AB “personnel turnover”) OR (TI “police turnover” OR AB “police turnover”) OR (TI “labo#r turnover” OR AB “labo#r turnover”) OR (TI staffing OR AB staffing) OR (TI “personnel downsizing” OR AB “personnel downsizing”) OR (TI “sickness absence*” OR AB “sickness absence*”) OR (TI absentee* OR AB absentee*) OR (TI “resource allocation” OR AB “resource allocation”) OR (TI “police resource*” OR AB “police resource*”) |  | 1,885,103 |
| S7 |  | S5 OR S6 |  | 1,975,730 |
| S8 | Mental Wellbeing Outcomes | (MH "Occupational Health") OR (MH "stress, Physiological") OR (MH "Mental Health") OR (MH "Mental Disorders") OR (MH "stress Disorders, Post-Traumatic") OR (MH "stress Disorders, Traumatic") OR (MH "Occupational Diseases") OR (MH "Burnout, Professional") OR (MH "Fatigue") OR (MH "Depression") OR (MH "Depressive Disorder") OR (MH "Anxiety") OR (MH "Anxiety Disorders") OR (MH "Personality Disorders") OR (MH "Adjustment Disorders") OR (MH "Neurotic Disorders") OR (MH "Suicide") OR (MH "Resilience, Psychological") OR (MH "Quality of Life") OR (MH "Morale") OR (MH "Depersonalization") |  | 751,545 |
| S9 |  | (TI “occupational health” OR AB “occupational health”) OR (TI “job stress*” OR AB “job stress*) OR (TI stress* OR AB stress*) OR (TI “occupational stress*” OR AB “occupational stress*”) OR (TI “psychological wellbeing” OR AB “psychological wellbeing”) OR (TI “psychological well-being” OR AB “psychological well-being”) OR (TI “mental wellbeing” OR AB “mental wellbeing”) OR (TI “mental well-being” OR AB “mental well-being”) OR (TI “mental disorder*” OR AB “mental disorder*”) OR (TI “psychological disorder*” OR AB “psychological disorder*”) OR (TI “mental health” OR AB “mental health”) OR (TI “psychological health” OR AB “psychological health”) OR (TI “mental illness*” OR AB “mental illness*”) OR (TI “psychological illness*” OR AB “psychological illness*”) OR (TI “work related illness*” OR AB “work related illness*”) OR (TI “occupational disease*” OR AB “occupational disease*”) OR (TI “stress disorder*” OR AB “stress disorder*”) OR (TI “stress related illness*” OR AB “stress related illness*”) OR (TI Burnout OR AB Burnout) OR (TI “Emotional Exhaustion” OR AB “Emotional Exhaustion”) OR (TI fatigue OR AB fatigue) OR (TI depress* OR AB depress*) OR (TI Anxiety OR AB Anxiety) OR (TI “Anxiety disorder*” OR AB “Anxiety disorder*”) OR (TI “cognitive disorder*” OR AB “cognitive disorder*”) OR (TI “personality disorder*” OR AB “personality disorder*”) OR (TI “psychotic disorder*” OR AB “psychotic disorder*”) OR (TI “adjustment disorder*” OR “adjustment disorder*”) OR (TI “emotional disorder*” OR AB “emotional disorder*”) OR (TI “neurotic disorder*” OR “neurotic disorder*”) OR (TI neurosis OR AB neurosis) OR (TI psychosis OR AB psychosis) OR (TI mania* OR AB mania*) OR (TI suicid* OR AB sucid*) OR (TI “self injur*” OR AB “self injur*”) OR (TI “self harm*” OR AB “self harm*”) OR (TI “post traumatic stress*” OR AB “post traumatic stress*”) OR (TI ptsd OR AB ptsd) OR (TI somatization OR AB somatization) OR (TI resilience OR AB resilience) OR (TI restless* OR AB restless*) OR (TI “quality of life” OR AB “quality of life”) OR (TI “quality of work life” OR AB “quality of work life”) OR (TI morale OR AB morale) OR (TI depersonali?ation OR AB depersonali?ation) OR (TI isolation OR AB isolation) OR (TI disengage* OR AB disengage*) OR (TI powerless* OR AB powerless*) OR (TI detach* OR AB detach*) OR (TI meaninglessness OR AB meaninglessness) OR (TI insecurit* OR AB insecurit*) OR (TI helpless* OR AB helpless*) |  | 296,637 |
| S10 |  | S8 OR S9 |  | 1,032,028 |
| S11 |  | S3 AND S4 AND S7 AND S10 | Limiters - Publication Year: 1990-2017; English; Language: English | 3 |
| S12 |  | S3 AND S7 AND S10 | Limiters - Publication Year: 1990-2017; English; Language: English | 759 |
| S13 |  | S11 OR S12 | Limiters - Publication Year: 1990-2017; English; Language: English | 759 |

# Table S5 EMBASE Search Strategy Database: EMBASE 1947-present Interface: OVID Research Database Search date: 16/05/2017 Search screen: Advanced Search; Search modes: Boolean/Phrase Medical subject heading (MeSH): /; Word in title or abstract: tw

| No. | PICO | Search Term | Limits applied | Hits |
| --- | --- | --- | --- | --- |
| S1 | Population | law enforcement/ or police/ |  | 18,124 |
| S2 |  | (police* or "law enforce*" or "blue coat*" or garda* or SWAT).tw. |  | 23,060 |
| S3 |  | 1 or 2 |  | 31,493 |
| S4 | Organisational Structure | organizational structure/ |  | 1,837 |
| S5 |  | ("organi?ational structure*" or "workplace structure*" or "work structure*" or "police structure*" or "rank structure*" or "organi?ational hierarch*" or "workplace hierarch*" or "work hierarch*" or "police hierarch*").tw. |  | 4069 |
| S6 |  | 4 or 5 |  | 5,211 |
| S7 | Organisational Stressors | workload/ or work environment/ or salary/ or job security/ or reward/ or conflict/ or violence/ or bullying/ or harassment/ or professional misconduct/ or leadership/ or criminal justice/ or absenteeism/ or resource allocation/ |  | 245,242 |
| S8 |  | ("organi?ational demand*" or "workplace demand*" or "work demand*" or "job demand*" or "work load*" or "workload*" or "work overload" or "heavy caseload*" or "work activit*" or "working hour*" or overtime or "job strain" or "work related strain" or "work control*" or "workplace control*" or "job control*" or "decision latitude" or "work pressure*" or "job pressure*" or "workplace pressure*" or "time pressure*" or "working condition*" or "physical danger*" or "work environment*" or "organi?ational environment*" or "organi?ational climate*" or "work climate*" or "organi?ational culture*" or "work culture*" or "police culture*" or "demand and control" or "demand control support" or "skill utili?ation" or "job skill*" or "professional worth" or "organi?ational communication*" or "role responsib*" or "police responsib*" or "police power*" or "role requirement*" or "role ambiguity" or "role problem*" or reward* or salar* or wage* or pay or "opportunit*" or "career develop*" or "promotion prospect*" or "career prospect*" or "career advance*" or ("employment insecurit*" or "job insecurit*" or "job security" or "employment securit*" or conflict* or violence or incivil* or uncivil or corrupt* or aggress* or misconduct or brutality or racism or unrest or cynic* or hostil* or bullying or harass* or discontent* or "blame culture*" or "interprofessional relation*" or "police manage*" or leader* or "supervisory influence*" or "support*" or "police supervision" or "police administration*" or consultation* or "occupational education" or "occupational training" or "organi?ational justice" or "organi?ational injustice" or "criminal justice*" or "personnel turnover" or "police turnover" or "labo#r turnover" or staffing or "personnel downsizing" or "sickness absence*" or absentee* or "resource allocation" or "police resource*")).tw. |  | 2,419,920 |
| S9 |  | 7 or 8 |  | 2,521,703 |
| S10 | Mental Wellbeing Outcomes | occupational health/ or stress/ or job stress/ or mental stress/ or psychological well-being/ or mental health/ or occupational disease/ or Anxiety/ or stress/ or posttraumatic stress disorder/ or Depression/ or Burnout/ or Mental Disorders/ or fatigue/ or Anxiety disorder/ or personality disorder/ or adjustment disorder/ or neurosis/ or mania/ or suicide/ or posttraumatic stress disorder/ or "quality of working life"/ or "quality of life"/ or depersonalization/ or helplessness/ |  | 1,341,751 |
| S11 |  | ("occupational health" or "job stress*" or stress* or "occupational stress*" or "psychological wellbeing" or "psychological well-being" or "mental wellbeing" or "mental well-being" or "mental disorder*" or "psychological disorder*" or "mental health" or "psychological health" or "mental illness*" or "psychological illness*" or "work related illness*" or "occupational disease*" or "stress disorder*" or "stress related illness*" or Burnout or "Emotional Exhaustion" or fatigue or depress* or Anxiety or "Anxiety disorder*" or "cognitive disorder*" or "personality disorder*" or "psychotic disorder*" or ("adjustment disorder*" or "emotional disorder*" or "neurotic disorder*" or neurosis or psychosis or mania* or suicid* or "self injur*" or "self harm" or "post traumatic stress" or ptsd or somatization or resilience or restless* or "quality of life" or "quality of work life" or morale or depersonali?ation or isolation or disengage* or powerless* or detach* or meaninglessness or insecurity or helpless*)).tw. |  | 2,365,915 |
| S12 |  | 10 or 11 |  | 2,827,881 |
| S13 |  | 3 and 6 and 9 and 12 |  | 10 |
| S14 |  | limit 13 to (english language and yr="1990 -Current") |  | 8 |
| S15 |  | 3 and 9 and 12 |  | 2,477 |
| S16 |  | limit 15 to (english language and yr="1990 - 2017") |  | 2,101 |
| S17 |  | 14 or 16 |  | 2,101 |

Table S6

SocINDEX Search Strategy

Database: SocINDEX with Full-Text (1895 to present date)

Interface: EBSCOhost Research Databases

Search date: 16/05/2017

Search screen: Advanced Search; Search modes: Boolean/Phrase

Exact thesaurus term: DE; Title: TI; Abstract: AB

| No. | PICO | Search Term | Limits applied | Hits |
| --- | --- | --- | --- | --- |
| S1 | Population | DE "POLICE" OR DE "LAW enforcement" |  | 18,761 |
| S2 |  | (TI police* OR AB police*) OR (TI “law enforce*” OR AB “law enforce*”) OR ( TI "blue coat*" OR AB "blue coat*" ) OR (TI garda* OR AB garda*) OR (TI SWAT OR AB SWAT) |  | 72,346 |
| S3 |  | S1 OR S2 |  | 75,497 |
| S4 | Organisational Structure | DE "WORK structure" |  | 337 |
| S5 |  | (TI "organi?ational structure*" OR AB "organi?ational structure*”) OR (TI "workplace structure*" OR AB "workplace structure*") OR (TI "work structure*" OR AB "work structure*") OR (TI "police structure*" OR AB "police structure*") OR (TI "rank structure*" OR AB "rank structure*") OR (TI "organi?ational hierarch*" OR AB "organi?ational hierarch*”) OR (TI "workplace hierarch*" OR AB "workplace hierarch*”) OR (TI "work hierarch*" OR AB "work hierarch*”) OR (TI "police hierarch*" OR AB "police hierarch*”) |  | 3,894 |
| S6 |  | S4 OR S5 |  | 4,213 |
| S7 | Organisational Stressors | DE "WORKING hours" OR DE "COMMUNICATION in organizations" OR DE "WAGES" OR DE "CAREER development" OR DE "JOB security" OR DE "JOB skills" OR DE "CONFLICT (Psychology)" OR DE "INTERPROFESSIONAL relations" OR DE "POLICE administration" OR DE "POLICE brutality" OR DE "POLICE corruption" OR DE "EMPLOYEES -- Workload" OR DE "POLICE harassment" OR DE "POLICE misconduct" OR DE "POLICE power" OR DE "POLICE racism" OR DE "WORK environment" OR DE "OCCUPATIONAL training" OR DE "ORGANIZATIONAL justice" OR DE "CRIMINAL justice administration" OR DE "LABOR turnover" OR DE "RESOURCE allocation" OR DE "REWARD (Psychology)" |  | 54,623 |
| S8 |  | (TI "organi?ational demand*" OR AB "organi?ational demand*”) OR (TI "workplace demand*" OR AB "workplace demand*”) OR (TI "work demand*" OR AB "work demand*”) OR (TI "job demand*" OR AB "job demand*”) OR (TI "work load*" OR AB "work load*”) OR (TI "workload*" OR AB "workload*”) OR (TI "heavy caseload*" OR AB "heavy caseload*”) OR (TI "work activit*" OR AB "work activit*") OR (TI "working hour*" OR AB "working hour*") OR (TI overtime OR AB overtime) OR (TI "job strain" OR AB "job strain”) OR (TI "work related strain" OR AB "work related strain”) OR (TI "work control*" OR AB "work control*”) OR (TI "workplace control*" OR AB "workplace control*”) OR (TI "job control*" OR AB "job control*”) OR (TI "decision latitude" OR AB "decision latitude") OR (TI "work pressure*" OR AB "work pressure*") OR (TI "job pressure*" OR AB "job pressure*") OR (TI "workplace pressure*" OR AB "workplace pressure*") OR (TI "time pressure*" OR AB "time pressure*") OR (TI “working condition*” OR AB “working condition*”) OR (TI "physical danger*" OR AB "physical danger*") OR (TI “work environment*” OR AB “work environment*”) OR (TI "organi?ational environment*" OR AB "organi?ational environment*") OR (TI "organi?ational climate*" OR AB "organi?ational climate*") OR (TI "work climate*" OR AB "work climate*") OR (TI "organi?ational culture*" OR "organi?ational culture*") OR (TI "work culture*" OR AB "work culture*") OR (TI "police culture*" OR AB "police culture*") OR (TI "demand and control" OR AB "demand and control") OR (TI "demand control support" OR AB "demand control support") OR (TI "skill utili?ation" OR AB "skill utili?ation") OR (TI "job skill*" OR AB "job skill*”) OR (TI "professional worth*" OR AB "professional worth*") OR (TI "organi?ational communication*" OR AB "organi?ational communication*") OR (TI "role responsib*" OR AB "role responsib*") OR (TI "police responsib*" OR AB "police responsib*") OR (TI "police power*" OR AB "police power*") OR (TI "role requirement*" OR AB "role requirement*") OR (TI "role ambiguity" OR AB "role ambiguity") OR (TI "role problem*" OR AB "role problem*") OR (TI reward* OR AB reward*) OR (TI salar* OR AB salar*) OR (TI pay OR AB pay) OR (TI wage* OR AB wage*) OR (TI " opportunit*" OR "opportunit*") OR (TI "career develop*" OR AB "career develop*") OR (TI "promotion prospect*" OR AB "promotion prospect*") OR (TI "career prospect*" OR AB "career prospect*") OR (TI “career advance*” OR AB “career advance*”) OR (TI “employment insecurit*” OR AB “employment insecurit*”) OR (TI “employment securit*” OR “employment securit*” ) OR (TI “job security” OR AB “job security”) OR (TI “job insecurit*” OR AB “job insecurit*”) OR (TI conflict* OR AB conflict*) OR (TI violence OR AB violence) OR (TI incivil* OR AB incivil*) OR (TI uncivil OR AB uncivil) OR (TI corrupt* OR AB corrupt*) OR (TI aggress* OR AB aggress*) OR(TI misconduct OR AB misconduct) OR(TI brutality OR AB brutality) OR(TI racism OR AB racism) OR (TI unrest OR AB unrest) OR(TI cynic* OR AB cynic*) OR (TI hostil* OR AB hostil*) OR (TI bullying OR AB bullying) OR (TI harass* OR AB harass*) OR (TI discontent* OR AB discontent*) OR (TI “blame culture*” OR AB “blame culture*”) OR (TI “interprofessional relation*” OR AB “interprofessional relation*”) OR (TI “police manage*” OR AB “police manage*”) OR (TI leader* OR AB leader*) OR (TI “supervisory influence*” OR AB “supervisory influence*”) OR (TI support* OR AB support*) OR (TI “police supervision” OR AB “police supervision”) OR (TI “police administration*” OR AB “police administration*”) OR (TI consultation* OR AB consultation*) OR (TI “occupational education” OR AB “occupational education”) OR (TI “occupational training” OR AB “occupational training”) OR (TI “organi?ational justice” OR AB “organi?ational justice”) OR (TI “organi?ational injustice*” OR AB “organi?ational injustice*”) OR (TI “criminal justice*” OR AB “criminal justice*”) OR (TI “personnel turnover” OR AB “personnel turnover”) OR (TI “police turnover” OR AB “police turnover”) OR (TI “labo#r turnover” OR AB “labo#r turnover”) OR (TI staffing OR AB staffing) OR (TI “personnel downsizing” OR AB “personnel downsizing”) OR (TI “sickness absence*” OR AB “sickness absence*”) OR (TI absentee* OR AB absentee*) OR (TI “resource allocation” OR AB “resource allocation”) OR (TI “police resource*” OR AB “police resource*”) |  | 528,635 |
| S9 |  | S7 OR S8 |  | 549,780 |
| S10 | Mental Wellbeing Outcomes | DE "JOB stress" OR DE "*STRESS* & disease" OR DE "MENTAL illness" OR DE "BURNOUT (Psychology)" OR DE "MENTAL health" OR DE "*ANXIETY*" OR DE "NEUROSES" OR DE "*ANXIETY* disorders" OR DE "MENTAL Depression" OR DE "PERSONALITY disorders" OR DE "SUICIDAL behavior" OR DE "QUALITY of life" OR DE "QUALITY of work life" OR DE "MORALE" OR DE "DEPERSONALIZATION" OR DE "HELPLESSNESS (Psychology)" |  | 54,943 |
| S11 |  | (TI “occupational health” OR AB “occupational health”) OR (TI “job stress*” OR AB “job stress*) OR (TI stress* OR AB stress*) OR (TI “occupational stress*” OR AB “occupational stress*”) OR (TI “psychological wellbeing” OR AB “psychological wellbeing”) OR (TI “psychological well-being” OR AB “psychological well-being”) OR (TI “mental wellbeing” OR AB “mental wellbeing”) OR (TI “mental well-being” OR AB “mental well-being”) OR (TI “mental disorder*” OR AB “mental disorder*”) OR (TI “psychological disorder*” OR AB “psychological disorder*”) OR (TI “mental health” OR AB “mental health”) OR (TI “psychological health” OR AB “psychological health”) OR (TI “mental illness*” OR AB “mental illness*”) OR (TI “psychological illness*” OR AB “psychological illness*”) OR (TI “work related illness*” OR AB “work related illness*”) OR (TI “occupational disease*” OR AB “occupational disease*”) OR (TI “stress disorder*” OR AB “stress disorder*”) OR (TI “stress related illness*” OR AB “stress related illness*”) OR (TI Burnout OR AB Burnout) OR (TI “Emotional Exhaustion” OR AB “Emotional Exhaustion”) OR (TI fatigue OR AB fatigue) OR (TI depress* OR AB depress*) OR (TI Anxiety OR AB Anxiety) OR (TI “Anxiety disorder*” OR AB “Anxiety disorder*”) OR (TI “cognitive disorder*” OR AB “cognitive disorder*”) OR (TI “personality disorder*” OR AB “personality disorder*”) OR (TI “psychotic disorder*” OR AB “psychotic disorder*”) OR (TI “adjustment disorder*” OR “adjustment disorder*”) OR (TI “emotional disorder*” OR AB “emotional disorder*”) OR (TI “neurotic disorder*” OR “neurotic disorder*”) OR (TI neurosis OR AB neurosis) OR (TI psychosis OR AB psychosis) OR (TI mania* OR AB mania*) OR (TI suicid* OR AB sucid*) OR (TI “self injur*” OR AB “self injur*”) OR (TI “self harm*” OR AB “self harm*”) OR (TI “post traumatic stress*” OR AB “post traumatic stress*”) OR (TI ptsd OR AB ptsd) OR (TI somatization OR AB somatization) OR (TI resilience OR AB resilience) OR (TI restless* OR AB restless*) OR (TI “quality of life” OR AB “quality of life”) OR (TI “quality of work life” OR AB “quality of work life”) OR (TI morale OR AB morale) OR (TI depersonali?ation OR AB depersonali?ation) OR (TI isolation OR AB isolation) OR (TI disengage* OR AB disengage*) OR (TI powerless* OR AB powerless*) OR (TI detach* OR AB detach*) OR (TI meaninglessness OR AB meaninglessness) OR (TI insecurit* OR AB insecurit*) OR (TI helpless* OR AB helpless*) |  | 24,413 |
| S12 |  | S10 OR S11 |  | 77,090 |
| S13 |  | S3 AND S6 AND S9 AND S12 | Limiters - Publication Year: 1990-2017; English; Language: English | 5 |
| S14 |  | S3 AND S9 AND S12 | Limiters - Publication Year: 1990-2017; English; Language: English | 343 |
| S15 |  | S13 OR S14 | Limiters - Publication Year: 1990-2017; English; Language: English | 343 |

Table S7

PsycINFO Search Strategy

Database: PsycINFO (1887-present day)

Interface: EBSCOhost Research Databases

Search date: 16/05/2017

Search screen: Advanced Search; Search modes: Boolean/Phrase

Exact thesaurus term: DE; Title: TI; Abstract: AB

| No. | PICO | Search Term | Limits applied | Hits |
| --- | --- | --- | --- | --- |
| S1 | Population | (DE "Police Personnel") OR (DE "Law Enforcement") OR (DE "Law Enforcement Personnel") |  | 11,595 |
| S2 |  | (TI police* OR AB police*) OR (TI “law enforce*” OR AB “law enforce*”) OR (TI "blue coat*" OR AB "blue coat*”) OR (TI garda* OR AB garda*) OR (TI SWAT OR AB SWAT) |  | 22,534 |
| S3 |  | S1 OR S2 |  | 23,347 |
| S4 | Organisational Structure | DE "Organizational Structure" |  | 5,927 |
| S5 |  | (TI "organi?ational structure*" OR AB "organi?ational structure*”) OR (TI "workplace structure*" OR AB "workplace structure*") OR (TI "work structure*" OR AB "work structure*") OR (TI "police structure*" OR AB "police structure*") OR (TI "rank structure*" OR AB "rank structure*") OR (TI "organi?ational hierarch*" OR AB "organi?ational hierarch*”) OR (TI "workplace hierarch*" OR AB "workplace hierarch*”) OR (TI "work hierarch*" OR AB "work hierarch*”) OR (TI "police hierarch*" OR AB "police hierarch*”) |  | 4,601 |
| S6 |  | S4 OR S5 |  | 9,098 |
| S7 | Organisational  Stressors | (DE "Work Load") OR (DE "Working Conditions") OR (DE "Organizational Climate") OR (DE "Rewards") OR (DE "Salaries") OR (DE "Career Development") OR (DE "Job Security") OR (DE "Conflict") OR (DE "Leadership") OR (DE "Workplace Violence") OR (DE "Criminal Justice") OR (DE "Employee Turnover") OR (DE "Employee Absenteeism") OR (DE "Resource Allocation") |  | 117,890 |
| S8 |  | (TI "organi?ational demand*" OR AB "organi?ational demand*”) OR (TI "workplace demand*" OR AB "workplace demand*”) OR (TI "work demand*" OR AB "work demand*”) OR (TI "job demand*" OR AB "job demand*”) OR (TI "work load*" OR AB "work load*”) OR (TI "workload*" OR AB "workload*”) OR (TI "heavy caseload*" OR AB "heavy caseload*”) OR (TI "work activit*" OR AB "work activit*") OR (TI "working hour*" OR AB "working hour*") OR (TI overtime OR AB overtime) OR (TI "job strain" OR AB "job strain”) OR (TI "work related strain" OR AB "work related strain”) OR (TI "work control*" OR AB "work control*”) OR (TI "workplace control*" OR AB "workplace control*”) OR (TI "job control*" OR AB "job control*”) OR (TI "decision latitude" OR AB "decision latitude") OR (TI "work pressure*" OR AB "work pressure*") OR (TI "job pressure*" OR AB "job pressure*") OR (TI "workplace pressure*" OR AB "workplace pressure*") OR (TI "time pressure*" OR AB "time pressure*") OR (TI “working condition*” OR AB “working condition*”) OR (TI "physical danger*" OR AB "physical danger*") OR (TI “work environment*” OR AB “work environment*”) OR (TI "organi?ational environment*" OR AB "organi?ational environment*") OR (TI "organi?ational climate*" OR AB "organi?ational climate*") OR (TI "work climate*" OR AB "work climate*") OR (TI "organi?ational culture*" OR "organi?ational culture*") OR (TI "work culture*" OR AB "work culture*") OR (TI "police culture*" OR AB "police culture*") OR (TI "demand and control" OR AB "demand and control") OR (TI "demand control support" OR AB "demand control support") OR (TI "skill utili?ation" OR AB "skill utili?ation") OR (TI "job skill*" OR AB "job skill*”) OR (TI "professional worth*" OR AB "professional worth*") OR (TI "organi?ational communication*" OR AB "organi?ational communication*") OR (TI "role responsib*" OR AB "role responsib*") OR (TI "police responsib*" OR AB "police responsib*") OR (TI "police power*" OR AB "police power*") OR (TI "role requirement*" OR AB "role requirement*") OR (TI "role ambiguity" OR AB "role ambiguity") OR (TI "role problem*" OR AB "role problem*") OR (TI reward* OR AB reward*) OR (TI salar* OR AB salar*) OR (TI pay OR AB pay) OR (TI wage* OR AB wage*) OR (TI "opportunit*" OR "opportunit*") OR (TI "career develop*" OR AB "career develop*") OR (TI "promotion prospect*" OR AB "promotion prospect*") OR (TI "career prospect*" OR AB "career prospect*") OR (TI “career advance*” OR AB “career advance*”) OR (TI “employment insecurit*” OR AB “employment insecurit*”) OR (TI “employment securit*” OR “employment securit*” ) OR (TI “job security” OR AB “job security”) OR (TI “job insecurit*” OR AB “job insecurit*”) OR (TI conflict* OR AB conflict*) OR (TI violence OR AB violence) OR (TI incivil* OR AB incivil*) OR (TI uncivil OR AB uncivil) OR (TI corrupt* OR AB corrupt*) OR (TI aggress* OR AB aggress*) OR(TI misconduct OR AB misconduct) OR(TI brutality OR AB brutality) OR(TI racism OR AB racism) OR (TI unrest OR AB unrest) OR(TI cynic* OR AB cynic*) OR (TI hostil* OR AB hostil*) OR (TI bullying OR AB bullying) OR (TI harass* OR AB harass*) OR (TI discontent* OR AB discontent*) OR (TI “blame culture*” OR AB “blame culture*”) OR (TI “interprofessional relation*” OR AB “interprofessional relation*”) OR (TI “police manage*” OR AB “police manage*”) OR (TI leader* OR AB leader*) OR (TI “supervisory influence*” OR AB “supervisory influence*”) OR (TI support* OR AB support*) OR (TI “police supervision” OR AB “police supervision”) OR (TI “police administration*” OR AB “police administration*”) OR (TI consultation* OR AB consultation*) OR (TI “occupational education” OR AB “occupational education”) OR (TI “occupational training” OR AB “occupational training”) OR (TI “organi?ational justice” OR AB “organi?ational justice”) OR (TI “organi?ational injustice*” OR AB “organi?ational injustice*”) OR (TI “criminal justice*” OR AB “criminal justice*”) OR (TI “personnel turnover” OR AB “personnel turnover”) OR (TI “police turnover” OR AB “police turnover”) OR (TI “labo#r turnover” OR AB “labo#r turnover”) OR (TI staffing OR AB staffing) OR (TI “personnel downsizing” OR AB “personnel downsizing”) OR (TI “sickness absence*” OR AB “sickness absence*”) OR (TI absentee* OR AB absentee*) OR (TI “resource allocation” OR AB “resource allocation”) OR (TI “police resource*” OR AB “police resource*”) |  | 1,011,184 |
| S9 |  | S7 OR S8 |  | 1,034,634 |
| S10 | Mental Wellbeing Outcomes | (DE "Occupational Health") OR (DE "Occupational stress") OR (DE "Mental Disorders") OR (DE "Mental Health") OR (DE "stress") OR (DE "Fatigue") OR (DE "Major Depression") OR (DE "Depression (Emotion)") OR (DE "Anxiety") OR (DE "Anxiety Disorders") OR (DE "Personality Disorders") OR (DE "Adjustment Disorders") OR (DE "Neurosis") OR (DE "Psychosis") OR (DE "Mania") OR (DE "Suicide") OR (DE "Post-Traumatic stress") OR (DE "Resilience (Psychological)") OR (DE "Quality of Life" ) OR (DE "Quality of Work Life") OR (DE "Morale") OR (DE "Depersonalization") OR (DE "Helplessness") |  | 524,100 |
| S11 |  | (TI “occupational health” OR AB “occupational health”) OR (TI “job stress*” OR AB “job stress*) OR (TI stress* OR AB stress*) OR (TI “occupational stress*” OR AB “occupational stress*”) OR (TI “psychological wellbeing” OR AB “psychological wellbeing”) OR (TI “psychological well-being” OR AB “psychological well-being”) OR (TI “mental wellbeing” OR AB “mental wellbeing”) OR (TI “mental well-being” OR AB “mental well-being”) OR (TI “mental disorder*” OR AB “mental disorder*”) OR (TI “psychological disorder*” OR AB “psychological disorder*”) OR (TI “mental health” OR AB “mental health”) OR (TI “psychological health” OR AB “psychological health”) OR (TI “mental illness*” OR AB “mental illness*”) OR (TI “psychological illness*” OR AB “psychological illness*”) OR (TI “work related illness*" OR AB “work related illness*”) OR (TI “occupational disease*” OR AB “occupational disease*”) OR (TI “stress disorder*” OR AB “stress disorder*”) OR (TI “stress related illness*” OR AB “stress related illness*”) OR (TI Burnout OR AB Burnout) OR (TI “Emotional Exhaustion” OR AB “Emotional Exhaustion”) OR (TI fatigue OR AB fatigue) OR (TI depress* OR AB depress*) OR (TI Anxiety OR AB Anxiety) OR (TI “Anxiety disorder*” OR AB “Anxiety disorder*”) OR (TI “cognitive disorder*” OR AB “cognitive disorder*”) OR (TI “personality disorder*” OR AB “personality disorder*”) OR (TI “psychotic disorder*” OR AB “psychotic disorder*”) OR (TI “adjustment disorder*” OR “adjustment disorder*”) OR (TI “emotional disorder*” OR AB “emotional disorder*”) OR (TI “neurotic disorder*” OR “neurotic disorder*”) OR (TI neurosis OR AB neurosis) OR (TI psychosis OR AB psychosis) OR (TI mania* OR AB mania*) OR (TI suicid* OR AB sucid*) OR (TI “self injur*” OR AB “self injur*”) OR (TI “self harm*” OR AB “self harm*”) OR (TI “post traumatic stress*” OR AB “post traumatic stress*”) OR (TI ptsd OR AB ptsd) OR (TI somatization OR AB somatization) OR (TI resilience OR AB resilience) OR (TI restless* OR AB restless*) OR (TI “quality of life” OR AB “quality of life”) OR (TI “quality of work life” OR AB “quality of work life”) OR (TI morale OR AB morale) OR (TI depersonali?ation OR AB depersonali?ation) OR (TI isolation OR AB isolation) OR (TI disengage* OR AB disengage*) OR (TI powerless* OR AB powerless*) OR (TI detach* OR AB detach*) OR (TI meaninglessness OR AB meaninglessness) OR (TI insecurit* OR AB insecurit*) OR (TI helpless* OR AB helpless*) |  | 61,659 |
| S12 |  | S10 OR S11 |  | 569,252 |
| S13 |  | S3 AND S6 AND S9 AND S12 | Limiters - Publication Year: 1990-2017; English; Language: English | 8 |
| S14 |  | S3 AND S9 AND S12 | Limiters - Publication Year: 1990-2017; English; Language: English | 1,414 |
| S15 |  | S13 AND S14 | Limiters - Publication Year: 1990-2017; English; Language: English | 1,414 |

1. Merriam-Webster. Police officer 2017 [Available from: <https://www.merriam-webster.com/dictionary/police%20officer>.
