# Supplementary material for: The relationship between organisational stressors and mental wellbeing within police officers: a systematic review
Source: BMC Public Health. 2019 Oct 15;19:1286. doi: 10.1186/s12889-019-7609-0 (PMC6792329; doi:10.1186/s12889-019-7609-0)
Supplement: Supplementary file 3 — Additional file 3: Table S8. PICO Screening Template (Table S8). [file 12889_2019_7609_MOESM3_ESM.docx]

The Relationship between Organisational Stressors and Mental Wellbeing within Police Officers: A Systematic Review

Additional File 3

File Format: DOC

Title: Table S8

Description: PICO Screening Template (Table S8)

Table S8

PICO Screening Template

| Review Question | | | The Relationship between Organisational Stressors and Mental Wellbeing within Police Officers: A Systematic Review | | | |
| --- | --- | --- | --- | --- | --- | --- |
| Reviewer Name | | |  | Date |  | |
| Study ID |  | Bibliographic citation |  | | | |
| Population | | Include | | Y/N | Exclude | Y/N |
|  | | Police personnel internationally from various ranks within police hierarchy of any age or gender, including trainees | |  | Correctional/prison/probation officers  Military/army/navy/forensic personnel  Police veteran’s  Civilian (Non-sworn police)  Traffic police  Other occupations |  |
| Exposure Variable(s) | | Include | | Y/N | Exclude | Y/N |
|  | | Stressors inherent in police work (I.e. work load, job demand, reward, opportunities, work pressure) | |  | Stressors not inherent in police work (i.e. personal stressors, family stressors) |  |
|  |  | Stressors arising internally from police department practices and policies (i.e.  leadership, supervision, support,  communication, culture,  climate, structure, hierarchy) | |  | External stressors stemming from society at large  (i.e. operational stressors) |  |
|  |  | External stressors stemming from the criminal justice system (i.e. organisational justice) | |  |  |  |
|  |  | Internal stressors confronting individual officers (i.e. corruption, incivility, bullying and violence) | |  |  |  |
| Outcome Variable(s) | | Must Include | | Y/N | Exclude | Y/N |
|  | | General mental health outcome (i.e. occupational stress, depression, anxiety and burnout) | |  | Only physiological wellbeing outcomes (i.e. blood pressure, cardiac autonomic control and salivary free cortisol response) | N |
|  |  | May Include | | Y/N | Organisational and personal outcomes (i.e. job satisfaction, job performance and job commitment) | Y |
|  |  | Subjective mental health outcome (i.e. helplessness, isolation, meaninglessness, insecurity, quality of life, quality of work life, morale, disengagement, powerlessness and detachment) | |  |  |  |
| Analysis | | Include | | Y/N | Exclude | Y/N |
|  | | Studies which have analysed the association between organisational stressors and mental wellbeing outcomes | |  | Descriptive studies which have assessed the prevalence of organisational stressors or mental wellbeing outcomes. |  |
| Study Design | | Include | | Y/N | Exclude | Y/N |
|  | | Published and non-published studies (accessed through contact with key authors), peer reviewed literature, doctoral dissertations, master’s theses and conference papers/reports | |  | Poster articles  Information piece (i.e. concept article, editorial) |  |
| Language | | Include | | Y/N | Exclude | Y/N |
|  | | Publications in English language | |  | Publications not in English |  |
| Time period | | Include | | Y/N | Exclude | Y/N |
|  | | Publications 1990 to present date | |  | Publications prior to 1990 |  |
| Overall Decision | | INCLUDED | |  | EXCLUDED |  |
| Notes | |  | | | | |
