# Supplementary material for: The relationship between organisational stressors and mental wellbeing within police officers: a systematic review
Source: BMC Public Health. 2019 Oct 15;19:1286. doi: 10.1186/s12889-019-7609-0 (PMC6792329; doi:10.1186/s12889-019-7609-0)
Supplement: Supplementary file 4 — Additional file 4: Table S9. Excluded Studies with Reason for Exclusion (Table S9). [file 12889_2019_7609_MOESM4_ESM.docx]

The Relationship between Organisational Stressors and Mental Wellbeing within Police Officers: A Systematic Review

Additional File 4

File Format: DOC

Title: Table S9

Description: Excluded Studies with Reason for Exclusion (Table S9)

Table S9

Excluded Studies with Reason for Exclusion

| Bibliographic Citation | Reason for Exclusion | Reason for Exclusion as Per PICO Statement |
| --- | --- | --- |
| Acquadro Maran D, Varetto A, Zedda M, Ieraci V. Occupational stress, anxiety and coping strategies in police officers. *Occupational Medicine* (Oxford, England). 2015; 65(6): 466-73. | Study assessed coping strategies on stress. | Inappropriate exposure |
| Agolla JE. Occupational stress among police officers: the case of Botswana police service. *Research Journal of Business Management*. 2009; 3:25-35. | Study graded frequency of organisational stressors and collated information on frequency of symptoms of stress. No formal statistical analysis was carried out investigating the association between stressors and symptoms of stress. | Descriptive study assessing organisational stressor prevalence |
| Almale BD, Vankudre AJ, Bansode-Gokhe SS, Pawar VK. An epidemiologic study of occupational stress factors in Mumbai police personnel. *Indian Journal of Occupational and Environmental Medicine*. 2014; 18(3): 109-12. | Study assessed the prevalence of organisational stressors. Unable to access tables detailing the findings from formal statistical analysis carried out. | Descriptive study assessing organisational stressor prevalence |
| Andrew ME, McCanlies EC, Burchfiel CM, Charles LE, Hartley TA, Fekedulegn D, *et al*. Hardiness and Psychological Distress in a cohort of police officers. *Int J Emerg Ment Health*. 2008; 10(2): 137-47. | Study failed to address organisational stressors; focus was on individual hardiness personality traits and their association with post-traumatic stress disorder (PTSD), depression, psychological symptoms and distress. | Inappropriate exposure |
| Aranda Beltrán C, Pando Moreno M, Salazar Estrada JG, Torres López TM, Aldrete Rodríguez MG. Social support, burnout syndrome and occupational exhaustion among Mexican traffic police agents. *The Spanish Journal Of Psychology*. 2009; 12(2): 585-92. | Support measure was a composite measure for both family and organisational support. An analysis, which considered organisational support measures and their association with psychological wellbeing could not be extracted. Study population was traffic police. | (1) Inappropriate population  (2) Inappropriate exposure |
| Bakker AB, Heuven E. Emotional dissonance, burnout, and in-role performance among nurses and police officers. *International Journal of stress Management.* 2006; 13(4): 423-40. | Study investigated the effect of individual emotional dissonance on Burnout and role performance. | Inappropriate exposure |
| Bano B. Job stress among police personnel. *International Conference on Economics and Finance Research.* 2011; 4:290-3. | Study focus was on the association between socio demographic factors and occupational stress. | Inappropriate exposure |
| Baruch-Feldman C, Brondolo E, Ben-Dayan D, Schwartz J. Sources of social support and burnout, job satisfaction, and productivity. *J Occup Health Psychol.* 2002; 7(1): 84-93. | Study population was traffic enforcement officers. | Inappropriate population |
| Bawa N, Kaur R. Occupational stress and burnout among police officers. *Indian Journal of Community Psychology*. 2011; 7(2): 362-72. | Study focus was association between self-efficacy, hardiness and coping strategies with burnout. | Inappropriate exposure |
| Biggam FH, Power KG, MacDonald RR, Carcary WB, Moodie E. Self-perceived occupational stress and distress in a Scottish police force. *Work Stress*. 1997; 11(2): 118-33. | Study examined the differences in the prevalence of stress symptoms according to the variables of rank, gender and working location. | Descriptive study assessing prevalence of mental wellbeing outcomes |
| Bishopp SA, Boots DP. General strain theory, exposure to violence, and Suicide Ideation among police officers: A gendered approach. *Journal of Criminal Justice.* 2014; 42(6): 538-48. | Intervention focus was traumatic/critical incident stress. | Inappropriate exposure |
| Boag-Munroe F. *Police Federation of England and Wales (PFEW) pay and morale survey 2016 headline statistics*. Surrey: Police Federation of England and Wales (PFEW); 2016. | Study did not consider mental health outcomes. | Inappropriate outcome |
| Bohacik D. *Assessing the effects of distributive justice and procedural justice on occupational stress.* US: ProQuest Information & Learning; 2009. | Population was comprised of both police personnel and homeland security; a separate analysis of the relationship between police personnel organisational stressors and mental wellbeing outcomes could not be extracted. | Inappropriate population |
| Brandt DE. Social distress and the police. *Journal of Social Distress & the Homeless*. 1993; 2(4): 305-13. | Study assessed prevalence of administrative, organisational and duty stressors. No formal statistical analysis of association between stressors and mental wellbeing outcomes. | Descriptive study assessing organisational stressor prevalence |
| Brough P, Biggs A. Occupational stress in police and prison staff. In: Brown JM, Campbell EA, Brown JM, Campbell EA, editors. *The Cambridge handbook of forensic psychology.* New York, NY, US: Cambridge University Press; 2010. p. 707-17. | Publication was an information piece (concept article). | Inappropriate study type |
| Brown J, Cooper C, Kirkcaldy B. Occupational stress among senior police officers. *Br J Psychol.* 1996;87(1):31-41. | Study population included traffic police. A separate analysis assessing the impact of organisational stressors on the mental health of other police officers within the study population (superintendents and chief superintendents) could not be extracted. | Inappropriate population |
| Brown J, Grover J. The role of moderating variables between stressor exposure and being distressed in a sample of serving police officers. *Personality and Individual Differences*. 1998; 24(2): 181-5. | Study intervention was operational/traumatic stressors. | Inappropriate exposure |
| Burke KJ. Well-being in protective services personnel: Organisational influences. *Australasian Journal of Disaster and Trauma Studies.* 2006;(2). | Study assessed the association between stress coping mechanisms and job satisfaction. | (1) Inappropriate outcome  (2) Inappropriate exposure |
| Burke RJ. Toward an understanding of psychological Burnout among police officers. *Journal of Social Behaviour & Personality.* 1993;8(3):425-38. | Study exposure variable(s) were composite measures of multiple organisational stressors and operational stressors. | Inappropriate exposure |
| Chan J. Chapter 5: Police stress and occupational culture. *Sociology of Crime, Law & Deviance.* 2007; 8:129-51. | Study assessed the impact of organisational change on police perceptions of their work and culture. | (1) Inappropriate outcome  (2) Inappropriate exposure |
| Chueh K-H, Yen C-F, Lu L, Yang M-S *et al*. Association between psychosomatic symptoms and work stress among Taiwan police officers. *The Kaohsiung Journal of Medical Sciences.* 2011; 27(4): 144-9. | Study outcome was a measure of stress based on a composite measure of family role and organisational stressors. A separate analysis of the relationship between organisational stressors and mental wellbeing outcomes could not be extracted. | Inappropriate outcome |
| Collins PA, Gibbs AC. stress in police officers: a study of the origins, prevalence and severity of stress-related symptoms within a county police force. *Occup Med (Lond).* 2003; 53(4): 256-64. | Study examined the sources of stress related symptoms and measured the prevalence of associated mental ill health. No formal statistical analysis was conducted to assess the association between sources of stress and the prevalence of mental ill health. | (1) Descriptive study assessing prevalence of mental wellbeing outcomes  (2) Descriptive study assessing organisational stressor prevalence |
| Consultancy AMR. *Scottish Police Authority/Police Scotland opinion survey 2015.* Glasgow: Axiom Consultancy (Scotland); 2015. | Study outcome was general wellbeing and not specific mental wellbeing outcomes. | Inappropriate outcome |
| de Mesquita Silveira N, Vasconcellos SJL, Cruz LP, Kiles RF, Silva TP, Castilhos DG, *et al*. Assessment of Burnout levels in a sample of police officers. *Revista de Psiquiatria do Rio Grande do Sul.* 2005; 27(2): 1-9. | Study assessed burnout prevalence between two police departments, with no specific analysis of organisational stressor impact. | Descriptive study assessing prevalence of mental wellbeing outcomes |
| Deb S, Chakraborty T, Chatterjee P, Srivastava N. Job-related stress, causal factors and coping strategies of traffic constables. Journal of the *Indian Academy of Applied Psychology*. 2008; 34(1): 19-28. | Study population was traffic police. | Inappropriate population |
| Deschamps F, Paganon-Badinier I, Marchand AC, Merle C. Sources and assessment of occupational stress in the police. *J Occup Health*. 2003; 45(6): 358-64. | Study assessed the role of demographic characteristics on occupational stress levels of police. | Inappropriate exposure |
| Dollard MF, Osborne K, Manning I. Organization–environment adaptation: a macro-level shift in modelling work distress and moral. Journal of Organizational Behaviour. 2013; 34(5): 629-647. | Study investigated how emotional job resources could moderate the impact of emotional job demands on distress. | Inappropriate exposure |
| Dollard MF, Tuckey MR, Dormann C. Psychosocial safety climate moderates the job demand–resource interaction in predicting workgroup distress. *Accident Analysis and Prevention*. 2012; 45:694-704 | Study investigated how a psychosocial safety climate may enable the safe utilisation of resources to reduce demands and therefore reduce workplace distress. | Inappropriate exposure |
| Dunn ML, Cahill-Canning E. Back on the beat. *Occup Health (Lond).* 2005; 57(6): 24-7. | Study assessed the impact of short-term psychotherapy on the reduction of depression, anxiety and post-traumatic stress disorder. | Inappropriate exposure |
| Elliot-Davies M, Houdmont J. *Officer demand, capacity and welfare survey descriptive statistics summary report: absence behaviours January 2017*. Surrey: Police Federation of England and Wales (PFEW); 2017. | Report focus was on the sickness absence behaviours of police in England and Wales. | Descriptive study assessing organisational stressor prevalence |
| Elliot-Davies M, Houdmont J. *Officer demand, capacity and welfare survey descriptive statistics summary report: accidents, violence and injuries* January 2017. Surrey: Police Federation of England and Wales (PFEW); 2017. | Report focus was the assessment of accidents, violence and injuries within police in England and Wales. | Descriptive study assessing organisational stressor prevalence |
| Elliot-Davies M, Houdmont J. *Officer demand, capacity and welfare survey descriptive statistics summary report: attitude and evaluation measures January 2017.* Surrey: Police Federation of England and Wales (PFEW); 2017. | Report focus was the assessment of morale and organisational justice within police in England and Wales. | Descriptive study assessing organisational stressor prevalence |
| Elliot-Davies M, Houdmont J. *Officer demand, capacity and welfare survey descriptive statistics summary report: capacity January 2017.* Surrey: Police Federation of England and Wales (PFEW); 2017. | Report focus was the assessment of staffing levels in police in England and Wales. | Descriptive study assessing organisational stressor prevalence |
| Elliot-Davies M, Houdmont J. *Office demand, capacity and welfare survey descriptive statistics summary report: demand January 2017.* Surrey: Police Federation of England and Wales (PFEW); 2017. | Report focus was the assessment of job demands and workload levels police in England and Wales. | Descriptive study assessing organisational stressor prevalence |
| Elliot-Davies M, Houdmont J. *Officer demand, capacity and welfare survey descriptive statistics summary report: mental health and wellbeing January 2017.* Surrey: Police Federation of England and Wales (PFEW); 2017. | Report focus was the assessment of the stress levels of police in England and Wales. | Descriptive study assessing prevalence of mental wellbeing outcomes |
| Elliot-Davies M, Houdmont J*. Officer demand, capacity and welfare survey descriptive statistics summary report organisational support: mental health and wellbeing January 2017*. Surrey: Police Federation of England and Wales (PFEW); 2017. | Report focus was the assessment of support levels provided to police in England and Wales. | Descriptive study assessing organisational stressor prevalence |
| Evans BJ, Coman GJ. General versus specific measures of occupational stress: An Australian police survey. *Stress Medicine*. 1993; 9(1): 11-20. | Study assessed the impact of personality variables on stress appraisals. | Inappropriate exposure |
| Garbarino S, Cuomo G, Chiorri C, Magnavita N. Association of work-related stress with mental health problems in a special police force unit. *BMJ Open*. 2013;3(7):no pagination | Study assessed the impact of critical incident stress on special police force mental wellbeing. | Inappropriate exposure |
| Garcia L, Nesbary DK, Gu J. Perceptual variations of stressors among police officers during an era of decreasing crime. *Journal of Contemporary Criminal Justice*. 2004; 20(1): 33-50. | Study assessed the prevalence of organisational and operational stressors during an extended period of relatively low crime rates in Boston. | Descriptive study assessing organisational stressor prevalence |
| Hart PM, Wearing AJ, Headey B. Police stress and well-being: Integrating personality, coping and daily work experiences. *Journal of Occupational and Organizational Psychology*. 1995; 68(2): 133-56. | Study assessed the association between police personality and coping strategies on psychological wellbeing. | Inappropriate exposure |
| Hayday S, Broughton A, Tyers C. *Managing sickness absence in the police service a review of current practices.* Suffolk: Health and Safety Executive; 2007. | Report focus was on reasons for sickness absence and the implementation of interventions to reduce absence in UK police. | Inappropriate outcome |
| Hem E, Berg AM, Ekeberg O. Suicide in police- A critical review. Suicide and Life Threatening Behaviour. 2001; 31(2): 224-233. | Review aimed to compare the level and variation of suicide risk of police officers with the general population. | Descriptive study assessing prevalence of mental wellbeing outcome |
| Hu Q, Schaufeli WB, Taris TW. How are changes in exposure to job demands and job resources related to burnout and engagement? A longitudinal study among Chinese nurses and police officers. *Stress and Health.* 2017:no pagination. | Exposure variable, job demands, was a composite score which took into account work-family interference (excluded exposure stressor). | Inappropriate exposure |
| Huddleston L, Stephens C, Paton D. An evaluation of traumatic and organizational experiences on the psychological health of New Zealand police recruits. *Work: Journal of Prevention, Assessment & Rehabilitation.* 2007; 28(3): 199-207. | Study assessed the impact of traumatic and organisational stressors on psychological distress. A separate analysis of the impact of organisational stress on psychological distress could not be extracted. | Inappropriate exposure |
| Hunter S, Boss D. *Examining stress levels of DSP enforcement personnel and intervention techniques phase II.* Madison: Wisconsin Department of Transportation Research, Development & Technology Transfer; 2005. Report No.: 0092-03-01. | Report assessed prevalence of stress and investigated the optimal way to deliver stress management programmes to sworn police personnel. | Descriptive study assessing organisational stressor prevalence |
| Kim JL, Wells W, Vardalis JJ, Johnson SK, Lim H. Gender difference in occupational stress: A study of the South Korean National Police Agency. *International Journal of Law, Crime & Justice.* 2016; 44:163-82. | Study explored whether the sources of perceived stress are different for male and female police personnel. | Inappropriate outcome |
| Kop N, Euwema M, Schaufeli W. Burnout, job stress and violent behaviour among Dutch police officers. *Work and Stress.* 1999; 13(4): 326-40. | Study investigated burnout as a predictor of police behaviour. | Inappropriate outcome |
| Kunst MJJ, Saan MC, Bollen LJA, Kuijpers KF. Secondary traumatic stress and secondary posttraumatic growth in a sample of Dutch police family liaison officers. *Stress and Health.* 2017:no pagination. | Study investigated the association between traumatic stress and posttraumatic stress. | Inappropriate exposure |
| Kutlu R, Civi S, Karaoglu O. The assessment of quality of life and depression among police officers. Turkiye Klinikleri *Journal of Medical Sciences.* 2009; 29(1): 8-15. | Study investigated the prevalence of Depression in sample of police officers and assessed quality of life. | Descriptive study assessing prevalence of mental wellbeing outcomes |
| Larned JG. Understanding police suicide. *The Forensic Examiner*. 2010; 19(3): 64-71. | Report was an information piece (concept article) on police suicide. | Inappropriate study type |
| Lawson KJ, Rodwell JJ, Noblet AJ. Mental health of a police force: Estimating prevalence of work-related depression in Australia without a direct national measure. *Psychol Rep.* 2012; 110(3): 743-52. | Study assessed the prevalence of work related depression in a sample of sworn police officers. | Descriptive study assessing prevalence of mental wellbeing outcomes |
| Lee JH, Kim I, Won JU, Roh J. Post-traumatic stress disorder and occupational characteristics of police officers in republic of Korea: A cross-sectional study. *BMJ Open.* 2016; 6(3): no pagination. | Study assessed the impact of traumatic stressors on prevalence of PTSD. | Inappropriate exposure |
| Lee SG, Kim I, Kim D. Workplace violence and depressive symptomatology among police officer. *Occupational and Environmental Medicine.* 2014; 71:A76. | Poster article | Inappropriate study type |
| Liberman AM, Best SR, Metzler TJ, Fagan JA, Weiss DS, Marmar CR. Routine occupational stress and psychological distress in police. *Policing: An International Journal of Police Strategies & Management*. 2002;25(2):421-39. | Exposure was routine occupational stressors, a composite measure which included personal stressors. | Inappropriate exposure |
| Lima EP, Assuncao AA. Prevalence and factors associated with Posttraumatic stress disorder (PTSD) in emergency workers: A systematic literature review. *Rev.* 2011; 14(2): 217-30. | Study was not published in English. | Full-text not in English |
| Lord VB. An impact of community policing: Reported stressors, social support, and strain among police officers in a changing police department. *Journal of Criminal Justice.* 1996; 24(6): 503-22. | Study outcome was strain, assessed via physiological responses to stress, propensity to leave the profession and lack of job involvement. | Inappropriate outcome |
| Luceño-Moreno L, García-Albuerne Y, Talavera-Velasco B, Martín-García J. stress in Spanish police force depending on occupational rank, sex, age and work-shift. *Psicothema.* 2016; 28(4): 389-93. | Study described the levels of work stress of study respondents, describing the perception of psychosocial risk these study participants suffer. | Descriptive study assessing organisational stressor prevalence |
| Ma CC, Andrew ME, Fekedulegn D, Gu JK, Hartley TA, Charles LE, *et al*. Shift work and occupational stress in police officers. *Safety and Health at Work.* 2015; 6(1): 25-9. | Study assessed the frequency of occupational stressors during different shift working hours. | Descriptive study assessing organisational stressor prevalence |
| Maguen S, Metzler TJ, McCaslin SE, Inslicht SS, Henn-Haase C, Neylan TC, *et al*. Routine work environment stress and PTSD symptoms in police officers. *Journal of Nervous and Mental Disease.* 2009; 197(10): 754-60. | Study assessed the impact of critical incident exposure on PTSD development. | Inappropriate exposure |
| Maia DB, Marmar CR, Henn-Haase C, Nóbrega A, Fiszman A, Marques-Portella C, *et al*. Predictors of PTSD symptoms in brazilian police officers: the synergy of negative affect and peritraumatic dissociation. *Revista Brasileira De Psiquiatria* (Sao Paulo, Brazil: 1999). 2011; 33(4): 362-6. | Study assessed the impact of traumatic stressors on PTSD development. | Inappropriate exposure |
| Malach-Pines A, Keinan G. stress and Burnout in Israeli border police. *International Journal of Stress Management.* 2006; 13(4): 519-40. | Study identified the causes of stress in order to examine patterns of coping. | Inappropriate outcome |
| Marchand A, Durand P. Psychological Distress, Depression, and Burnout: Similar contribution of the job demand-control and job demand-control-support models. *Journal of Occupational and Environmental Medicine.* 2011;53(2):185-9. | Population comprised of both police officers and civilian staff. | Inappropriate population |
| Martin M, Marchand A, Boyer R, Martin N. Predictors of the development of posttraumatic stress disorder among police officers. *Journal of Trauma & Dissociation: The Official Journal of the International Society for The Study of Dissociation (ISSD).* 2009; 10(4): 451-68. | Study assessed the impact of traumatic stressors on PTSD development. | Inappropriate exposure |
| Masilamani R, Bulgiba A, Chinna K, Darus A, Isahak M, Kandiben S, *et al*. Prevalence and associated factors of stress in the Malaysian Police Force. *Preventive Medicine: An International Journal Devoted to Practice and Theory*. 2013; 57(Suppl): S57-S9. | Study estimated the prevalence of stress and investigated the impact of socio-demographic factors on stress levels (age, marital status, place of residence). | Descriptive study assessing prevalence of mental wellbeing outcomes |
| Mayes BT, Barton ME, Ganster DC. An exploration of the moderating effect of age on job stressor employee strain relationships. *Journal of Social Behavior & Personality*. 1991; 6(7): 289-308. | Study assessed the impact of ageing on study participants reaction to job stressors. | Inappropriate exposure |
| McCreary DR, Thompson MM. *The development of a reliable and valid measure of stressors in policing.* Toronto: Defence R & D Canada; 2004. | Report aimed to elicit the stressors associated with policing in order to develop and validate an operational and organisational police stress questionnaire. | Inappropriate outcome |
| Newman DW, Rucker-Reed ML. Police stress, state-trait anxiety, and stressors among U.S. Marshals. *Journal of Criminal Justice.* 2004; 32(6): 631-341. | Study investigated the association between personal variables and Anxiety in a sample of US marshals. | Inappropriate exposure |
| Ojedokun O, Balogun SK. The costs of policing: Psychosocial capital and mental health outcomes in a Nigeria police sample. *The Spanish Journal of Psychology.* 2015; 18. | Study investigated the impact of psychological capital on mental wellbeing outcomes. | Inappropriate exposure |
| Oweke JA, Muola J, Ngumi O. Causes of occupational stress in relation to level of occupational stress among police constables in Kisumu County, Kenya. *IOSR Journal of Humanities and Social Sciences.* 2014;19(11):13-20. | Study assessed prevalence of occupational stressors among police constables. | Descriptive study assessing organisational stressor prevalence |
| Padyab M, Backteman-Erlanson S, Brulin C. Burnout, coping, stress of conscience and psychosocial work environment among patrolling police officers. *Journal of Police and Criminal Psychology.* 2016;31(4):229-37. | Study focus was on the means by which stress related to troubled conscience can exacerbate individual response to work stressors. | Inappropriate exposure |
| Randall C, Buys N. Managing occupational stress injury in police services: A literature review. In: Sun J, Buys N, Merrick J, Sun J, Buys N, Merrick J, editors. *Health promotion: strengthening positive health and preventing disease. Health and human development.* Hauppauge, NY, US: Nova Biomedical Books; 2013. p. 49-70. | Review focus was on the management of occupational stress within the police force. | Inappropriate outcome |
| Riggins ES. *Career goals for joining law enforcement and subsequent career stress.* US: ProQuest Information & Learning; 2016. | Thesis explored the impact on individual stress when study participants could not accomplish personal goals in the work environment. | Inappropriate exposure |
| Schaible LM. *The impact of emotional labor, value dissonance, and occupational identity on police officers' levels of cynicism and burnout.* US: ProQuest Information & Learning; 2006. | Thesis explored the association between personal factors such as emotional management and value dissonance with cynicism and burnout | Inappropriate exposure |
| Scoggins SE, O’brien KJ. China’s unhappy police. *Asian Survey*. 2016; 56(2): 225-42. | Study investigated the association between heavy caseload, administrative drudgery and low pay on feelings of discontent, shirking, corruption and waste. | Inappropriate outcome |
| Sewell JD. Traumatic stress of multiple murder investigations. *J Trauma Stress.* 1993; 6(1): 103-18. | Study investigated the impact of operational traumatic stressors on police stress and the requirement for specific stress management programmes. | Inappropriate exposure |
| Shane J. Organizational stressors and police performance. *Journal of Criminal Justice*. 2010; 38(4): 807-18. | Study investigated the impact of co-worker relations, lack of training and resources, leadership and supervision, bureaucracy, internal affairs and accountability and management on police performance. | Inappropriate outcome |
| Singh S, Kar SK. Sources of occupational stress in the police personnel of North India: An exploratory study. *Indian Journal of Occupational and Environmental Medicine*. 2015; 19(1): 56-60. | Study compared the prevalence of organisational stressors between three ranks within the police hierarchy (inspectors, officers and constables). | Descriptive study assessing organisational stressor prevalence |
| Skogstad M, Skorstad M, Lie A, Conradi HS, Heir T, Weisaeth L. Work-related post-traumatic stress disorder. *Occup Med.* 2013; 63(3): 175-82. | Study explored stressors experienced by different occupational groups and the association with PTSD (police officers, ambulance personnel and fire fighters). The component of the review focused on police officers, considered the association between traumatic stressors and PTSD. | Inappropriate exposure |
| Stanfeld SA, Head J, Rasul F, Singleton N, Lee A. *Occupation and mental health: secondary analyses of the ONS psychiatric morbidity survey of Great Britain.* Suffolk: Health and Safety Executive; 2003. | Study explored the prevalence of common mental disorder between different occupational groups. | Descriptive study assessing prevalence of mental wellbeing outcomes |
| Stearns GM, Moore RJ. The physical and psychological correlates of job burnout in the Royal Canadian mounted police. *Canadian Journal of Criminology*. 1993; 35(2): 127-47. | Study did not consider the impact of organisational stressors on police mental wellbeing. | Inappropriate exposure |
| Storch JE, Panzarella R. Police stress: state-trait anxiety in relation to occupational and personal stressors. *Journal of Criminal Justice.* 1996; 24(2): 99-107. | Study investigated the association between personal stressors and operational task related stressors with state trait Anxiety. | Inappropriate exposure |
| Summerlin Z, Oehme K, Stern N, Valentine C. Disparate levels of stress in police and correctional officers: preliminary evidence from a pilot Study on domestic violence. *J Hum Behav Soc Environ*. 2010; 20(6): 762-77. | Study compared stress levels between police and correctional officers following an initiative to prevent officer involved domestic violence. | Inappropriate exposure |
| Suresh RS, Anantharam RN, Angusamy A, Ganesan J. Sources of job stress in police work in a developing country. *International Journal of Business and Management.* 2013;8(13): 102-110. | Study identified the job events perceived as significant sources of stress in police work, no formal statistical analysis was carried out on the association between job events and perceived levels of stress. | Descriptive study assessing organisational stressor prevalence |
| Toch H. *Stress in policing.* Washington, DC, US: American Psychological Association; 2002. | Report focus was on the identification of main sources of stress in policing. | Descriptive study assessing organisational stressor prevalence |
| Tomei G, Cherubini E, Ciarrocca M, Biondi M, Rosati MV, Tarsitani L, *et al*. Short communication: assessment of subjective stress in the municipal police force at the start and at the end of the shift. *Stress and Health.* 2006; 22(4): 239-47. | Study focus was the assessment of Anxiety, Depression, lack of social support, somatization, aggressiveness and total stress at the start and end of a police shift, comparing traffic police officers with a control group of police office based officers. | Descriptive study assessing prevalence of mental wellbeing outcomes |
| Tuckey MR, Dollard MF, Saebel J, Berry NM. Negative workplace behaviour: temporal associations with cardiovascular outcomes and psychological health problems in Australian police. *Stress and Health: Journal of the International Society for the Investigation of Stress.* 2010; 26(5): 372-81. | Study assessed the risk of poor mental and cardiovascular health following exposure to negative behaviour in the workplace. | Inappropriate exposure |
| UNISON. *2014 police staff stress survey report.* London: UNISON; 2014. | Report explored occupational stressors in policing and their prevalence in the UK. | Descriptive study assessing organisational stressor prevalence |
| Van Der Velden PG, Rademaker AR, Vermetten E, Portengen MA, Yzermans JC, Grievink L. Police officers: A high-risk group for the development of mental health disturbances? A cohort study. *BMJ Open.* 2013; 3(1): no pagination. | Study explored the prevalence of depression and anxiety in two groups of police officers compared to other occupational groups including bankers. | Descriptive study assessing prevalence of mental wellbeing outcomes |
| Van Dijk A, Crofts N. Law enforcement and public health as an emerging field. *Policing & Society.* 2017; 27(3): 261-75. | Study focus was on the integration of Public health and law enforcement. | Inappropriate outcome |
| Violanti JM, Andrew ME, Mnatsakanova A, Hartley TA, Fekedulegn D, Burchfiel CM. Correlates of hopelessness in the high suicide risk police occupation. *Police Practice & Research: An International Journal*. 2016; 17(5): 408-19. | Study analysed the association between organisational stressors and hopelessness (subjective mental wellbeing outcome). | Inappropriate outcome |
| Violanti JM, Fekedulegn D, Andrew ME, Charles LE, Hartley TA, Vila B, *et al*. Shift work and the incidence of injury among police officers. *Am J Ind Med.* 2012; 55(3): 217-27. | Study analysed the association between injury incidence among police officers and shift work. | Inappropriate outcome |
| Wang Y, Zheng L, Hu T, Zheng Q. Stress, burnout, and job satisfaction: case of police force in China. *Public Personnel Management.* 2014; 43(3): 325-39. | Study focus was on the mediating role of burnout and locus of control in the relationship between job stress and job satisfaction. | Inappropriate outcome |
| Wiegand DM, Brown SM. *Health hazard evaluation report: evaluation of job stress and morale at a federal law enforcement agency’s district facilities.* Cincinnati, OH: US Department of Health and Human Services, Centers for Disease Control and Prevention, National Institute for Occupational Safety and Health; 2014. Report No.: 2012-0160-3213. | Report assessed employee’s levels of job stress, morale, organisational commitment, job satisfaction, and mental health symptoms, work-related health concerns and perceptions of communication. No formal statistical analysis was conducted investigating the association between organisational stressors and mental health symptoms. | Descriptive study assessing prevalence of mental wellbeing outcomes |
| Wang Z, Inslicht SS, Metzler TJ, Henn-Haase C, McCaslin SE, Tong H, *et al*. A prospective study of predictors of depression symptoms in police. *Psychiatry Research.* 2010;175(3):211-6. | Exposure variable of interest (work stress) was a composite measure, which included personal stressors. | Inappropriate exposure |
| Žukauskas G, Rukšenas O, Burba B, Grigaliuniene V, Mitchell JT. A study of stress affecting police officers in Lithuania. *Int J Emerg Ment Health*. 2009; 11(4): 205-14. | Publication retracted | Publication retracted |

*Note.* Those studies with 2 or more reasons for exclusion are presented below in the format, reason (1), reason (2) etc. Reason (1) was considered the primary reason for exclusion and was the reason presented in the PRISMA flow diagram.
