# Supplementary material for: The relationship between organisational stressors and mental wellbeing within police officers: a systematic review
Source: BMC Public Health. 2019 Oct 15;19:1286. doi: 10.1186/s12889-019-7609-0 (PMC6792329; doi:10.1186/s12889-019-7609-0)
Supplement: Supplementary file 5 — Additional file 5: Tables S10 and S11. Results of Modified NOS Risk of Bias Assessment for all Included Studies (Table S10) and Summary of Included Studies (Table S11). [file 12889_2019_7609_MOESM5_ESM.docx]

The Relationship between Organisational Stressors and Mental Wellbeing within Police Officers: A Systematic Review

Additional File 5

File Format: DOC

Title: Table S10 and S11

Description: Results of Modified NOS Risk of Bias Assessment for all Included Studies (Table S10) and

Summary of Included Studies (Table S11)

Table S10

Results of Modified NOS Risk of Bias Assessment for all Included Studies

| *Study (n= 15)* | Study  Reference | Adams *et al*.(1) | Adebayo *et al*.(2) | Arial *et al*.(3) | Backteman-Erlanson *et al*.(4) | Berg *et al*.(5) | Chen *et al*.(6) | Crank *et al*. (7) | Houdmont *et al.*(8) | Janzen *et al*.(9) | Martinussen *et* *al*.(10) | McCarty *et al*.(11) | Morash *et al*.(12) | Morash *et al*.(13) | Mostert *et al*.(14) | Xavier *et al*.(15) |
| --- | --- | --- | --- | --- | --- | --- | --- | --- | --- | --- | --- | --- | --- | --- | --- | --- |
| Criterion | |  | | | | | | | | | | | | | | |
| A | Selection (Maximum 5 stars) |  | | | | | | | | | | | | | | |
|  | Representativeness of the sample (maximum 1 star) | * | * | * | * | * | * | * | * | * | * | * | * |  | * |  |
|  | Sample size (maximum 1 star) | * | * | * | * | * | * | * | * | * | * | * | * | * | * |  |
|  | Non-respondents (maximum 1 star) |  |  |  |  |  |  |  |  |  |  |  |  |  |  |  |
|  | Ascertainment of the exposure (risk factor) (maximum 2 stars) | ** | ** | * | ** | ** | ** | ** | ** | ** | ** | ** | ** | ** | ** |  |
| B | Comparability (Maximum 2 stars) |  | | | | | | | | | | | | | | |
|  | Subjects in different outcome groups are comparable based on study design or analysis. Confounding factors are controlled (maximum 2 stars) | * | * | ** | * | ** | * | * | * | * | ** |  | ** | ** | ** |  |
| C | Outcome (Maximum 3 stars) |  | | | | | | | | | | | | | | |
|  | Assessment of outcome (maximum 2 stars) | * | * | * | * | * | * | * | * | * | * | * | * | * | * | * |
|  | Statistical test (maximum 1 star) | * | * | * | * | * | * | * | * | * | * | * | * | * | * | * |
| D | Overall score (Maximum total 10 stars) | 7 | 7 | 7 | 7 | 8 | 7 | 7 | 7 | 7 | 8 | 6 | 8 | 7 | 8 | 2 |
|  | Ranking (High, intermediate and low quality) | High | High | High | High | High | High | High | High | High | High | Intermediate | High | High | High | Low |

Table S11

Summary of Included Studies

| Study ID |  | Exposure variable(s)^a^ | Outcome variable | Exposure instrumentation and  Cronbach’s alpha (α) | Outcome instrumentation and  Cronbach’s alpha (α) | Statistical analysis and measure of effect | Main findings | Adjustment by confounders (yes/no) | NOS grade |
| --- | --- | --- | --- | --- | --- | --- | --- | --- | --- |
| Crank *et al*. (7) |  | Personnel relations  Department issues | Occupational stress | Scale with 5 composite indexes to measure legitimacy issues. Personnel relations, 3-item subscale (α=.93); department issues 3-item subscale (α=.69) | 6-item index developed by (16) (α=.79) | Linear regression:  un-standardised beta coefficient (b) | Occupational stress significantly associated with department issues (b=.197, F=14.42, p<.05) but not personnel relations (b=.55, F=1.65, p= *ns*) | No | High |
| Morash *et al*. (12) |  | Lack of advancement opportunity  Ridicule and set ups  Lacks influence  Bias  Language harassment  Sexual harassment  Work group support | Occupational stress | Workplace problems scale developed and validated by Morash and Haarr (17). Lack of advancement opportunity, 3-item subscale (α=.73); ridicule and set ups 5-item subscale (α=.73); lacks influence, 4-item subscale (α.75); bias, 5-item subscale (α=.79); language harassment, 3-item subscale (α=.81); sexual harassment, 8-item subscale (α=.92)  Work group support scale consisted of 9 items (α=.73) adapted from (18). | 4-item occupational stress scale developed by Morash *et al*.(12) (α=.78) | Ordinary least squares regression:  standardised beta coefficient (β) | Occupational stress significantly associated with ridicule and set ups (β=.12, p<.01), lacks influence (β=.18, p<.01), bias (β=.29, p<.01), language harassment (β=.10, p<.01), sexual harassment (β=-.08, p<.05), but not lack of advancement opportunity (β=.03, p= *ns*) or work group support (β=0.04, p= *ns*) | Yes: exposure variables^a^ stated, physical overestimated, physical underestimated, invisible, stigma and appearance, family support, violent crime rate, property crime rate, token sex, token race, token ethnicity, type of agency, sex, minority, education, age and class when growing up | High |
| Morash *et al*. (13) |  | Sexual harassment  Language harassment  Ridicule and set ups  Superiors support | Occupational stress | Workplace problem scale developed and validated by Morash and Harr (17). Ridicule and set ups, 5-item subscale (α=.73); sexual harassment, 8-item subscale (α=.97); language harassment, 3-item subscale (α=0.9).  Support measures adapted from (19). Superiors support, 3-item subscale (α=.71) | 3-item occupational stress scale developed by Morash *et al*. (13) (α=.65) | Ordinary least squares regression:  standardised beta coefficient (β) | Occupational stress, significantly associated with ridicule and set-ups (β =.53, p <.01) and superiors support (β=-.26, p<.01), but not sexual harassment (β=.01, p= *ns*) or language harassment (β =.07, p= *ns*) | Yes: exposure variables stated^a^, feeling invisible, women and work, public disrespect, expressed feeling, changed my job, get others to like me, keep written records, stay in a group, age, sex, education, social class, married, length of service and rank | High |
| Berg *et al*. (5) |  | Job pressure  Lack of support | Anxiety | 30-item Job Stress Survey (JSS) (20).Job pressure, 10-item subscale (α=.83); lack of support, 10-item subscale (α=.83) | 14-item Hospital Anxiety and Depression Scale (HADS) (21). Anxiety, 7-item subscale (α= not reported); Depression, 7-item subscale (α= not reported) | Logistic regression: adjusted OR and 95% CI | Job pressure (OR 2.0, 95% CI:1.5-2.7; p<.001) was a significant predictor of Anxiety symptoms however lack of support (OR 1.2, 95% CI: 0.9-1.7; p= *ns*) was not | Yes: anxiety, depression, EE, DP, PA, subjective health complaints, suicidal ideation, age and gender | High |
| Berg *et al*. (5) |  | Job pressure  Lack of support | Depression | 30-item JSS (20). Job pressure, 10-item subscale (α=.83); lack of support, 10-item subscale (α=.83) | 14-item HADS Scale (21). Anxiety, 7-item subscale (α= not reported); Depression, 7-item subscale (α= not reported) | Logistic regression: adjusted OR and 95% CI | Job pressure (OR 1.0, 95% CI: 0.7-1.4, p= *ns*) and lack of support (OR 1.3, 95% CI: 0.9-1.99, p= *ns*) were not significantly associated with depression | Yes: anxiety, depression, subjective health complaints, suicidal ideation, age and gender | High |
| Chen *et al*. (6) |  | Judgements from peers  Heavy workload | Depression | Modified version of Disaster Related Psychological Screening Test (DRPST) (22) . Job stress, 3-item subscale, (α= not reported) | DRPST (22). Depression, 3-item subscale selected according to DSM-IV criteria of a major depressive episode (α= not reported) |  | Judgement from peers (OR 2.35, 95% CI:1.31-4.23, p=.004) and heavy workload (OR 1.73, 95% CI: 1.19-2.50, p=.004) as significant predictors of depression | Yes: exposure variables stated^a^, little time to spend with families, disharmony of marriage, child upbringing problems and job performance | High |
| Adams *et al*. (1) |  | Internal social stressors | PD | Internal social stressors measured by 7-item Workplace Incivility Scale (23) (α=.88) | PD measured by 13-item scale (24) (α=.92) | Hierarchical regression: standardised beta coefficients (β) | Insider social stressors positively correlated with PD (r=.57, p <.01). Insider social stressors and outsider social stressors accounted for 42 % of the variance in PD (R^2^=.42, p <.01). Insider social stressors were significantly associated with PD (β=.45, p <.01) when controlling for outsider social stressors. | Yes: outsider social stressors | High |
| Arial *et al*. (3) |  | High mental/  intellectual demand  Lack of support from superior and organisation  Inadequate work schedule | PS | Single items were used to measure stressors, deemed appropriate for face validity matters(3). No internal consistencies were reported. | Adapted version of Langner’s scale of Psychiatric Symptoms  (25) (26). Internal consistency from previous study  (27) reported (α=.77). | Multiple logistic regression: adjusted OR and 95% CI and standardised beta coefficients (β) | Lack of support from superior and organisation (OR 3.58, 95% CI:1.58–8.13) (β=1.28, p=.002), inadequate work schedule (OR 2.84, 95% CI: 1.22–6.62) (β =1.04, p=.016), high mental/intellectual demand (OR 2.56, 95% CI:1.12–5.86) (β= .94, p=.026), age in decades (OR 1.82, 95% CI:1.21–2.73)(β =.60, p=.004), Score for physical environment complaints (standardised, possible values 0-10) (OR 1.30, 95% CI: 1.03–1.64) (β=2.61, p=.028) were all significantly associated with PS at p<.05 significance level | Yes: exposure variables stated^a^, self-perception of bad quality work, age in decades and physical environment complaints | High |
| Houdmont *et al.* (8) |  | ≥ 49h/week = long working hours | PD | Respondents asked how many hours they worked in a typical week, excluding overtime, plus the number of hours of overtime. Responses were summed to produce overall weekly working hours (α= not reported) | 12-item General Health Questionnaire (GHQ-12)(28) used to measure PD (α=not reported) | Binary logistic regression:  adjusted OR and 95% CI | 52% (*n*= 637) of respondents demonstrated PD case-ness. The odds of PD case-ness in the ≥49-h group were double that of officers in the ≤48-h group following full adjustment (OR 2.05, 95%CI: 1.57–2.68, p<.05) | Yes: age, gender, rank, departmental tenure and years of police service | High |
| Janzen *et al*. (9) |  | Effort reward imbalance  Overcommitment | PD | 23-item Effort Reward Imbalance Questionnaire(29). Effort, 6 item subscale (α=.81); reward, 11-item subscale (α=.84); overcommitment, 6-item subscale (α=.85) | K6(30), 6-item subscale of nonspecific mental distress (α=.81) | Ordinary least squares regression: standardised beta coefficients (β) | Higher ‘effort-reward imbalance’ (β=.24, p<.05) and overcommitment’ (β=.40, p <.01) were significantly associated with greater PD | Yes: exposure variables stated^a^, age, gender, marital status (partnered) and education (university degree) | High |
| Xavier *et al*. (15) |  | Administrative/  organisational pressure  Police stress | Burnout | Police Stress Survey (PSS)  (α= not reported) | Maslach Burnout Inventory-Human Service Survey (MBI-HSS)  (α= not reported) | Correlation= (r) | Correlation demonstrated ‘police stress’ (r=.301 p<.01,1-tailed) and ‘administrative/organisational pressure’ (r=.347, p<.01, 1-tailed) were significantly correlated with burnout | No | Low |
| Adams *et al*. (1) |  | Internal social stressors | EE | Internal social stressors measured by 7-item Workplace Incivility Scale (23) (α=.88)  (23) | Wharton’s (31) 6-item scale of job-related EE (α=.88) | Hierarchical regression: standardised beta coefficients (β) | Internal social stressors and outsider social stressors’ together accounted for 46% of the variance in EE (R^2^ =.46, p <.01), Insider social stressors was significantly associated with EE (β=.44, p<.01) when controlling for outsider social stressors | Yes: outsider social stressors | High |
| Adebayo *et al*. (2) |  | Perceived workplace fairness | EE | 6-item, 7-point response scale (totally disagree to totally agree) originally developed by Van Yperen(32) (α=.82) | 9-item EE subscale from Maslach and Jackson(33) (α=.75) | Hierarchical multiple regression:  Standardised beta coefficients (β) | Perceived workplace fairness was significantly related to EE among the rank-and-file of Nigeria police (β=-.23, p<.01). As perceived level of workplace fairness increased, reported level of EE decreased | Yes: age, rank, education, tenure and gender | High |
| Backteman-Erlanson *et al*. (4) |  | Organisational culture  Organisational climate  Leadership  Demand  Decision latitude  Social support at work | EE | Dallner’’s QPSNordic questionnaire(34) which has 118 items, divided among 26 subscales measured organisational culture, 6-item subscale; organisational climate, 5-item subscale; leadership, 8 item subscale. For the three subscales (α=.71-.78)  Karasek and Theorell’s (35) demand-control questionnaire measured demand, five item subscale (α=.78); decision latitude, 6-item subscale (α=0.53); social support at work, 6-item subscale (α=.83) | A Swedish version of the MBI (36-38) assessed EE via 9-item subscale (α=.85). | Univariate logistic regression: univariate adjusted OR and 95% CI by gender | Organisational culture was significantly associated with EE in both females (OR 2.28, 95%CI: 1.61-3.21, p<.05) and male police (OR 2.09, 95%: 1.44-3.04, p<.05). As did organisational climate in both female (OR 2.48, 95% CI: 1.79-3.45, p<.05) and male police (OR 2.17, 95% CI: 1.56-3.01, p<.05). Social support was significantly associated with both female (OR 2.79, 95% CI:1.73- 4.51, p<.05), and male police (OR 3.47, 95% CI: 2.02-5.96, p<.05). Demand demonstrated a significant association with EE in both female (OR 7.69, 95% CI: 4.21-14.03, p<.05) and male police (OR 5.97, 95% CI: 3.32-10.71, p<.05). As did decision latitude in both female (OR 2.44, 95% CI:1.38-4.30, p<.05) and male police (OR 3.94, 95% CI:2.02-7.70, p<.05). Leadership in both female (OR 0.56, 95% CI:0.42-0.75, p=*ns*) and male police (OR 0.72, 95% CI:0.53-0.99, p=*ns*) did not demonstrate a significant association with EE | Yes: age | High |
| Berg *et al*. (5) |  | Job pressure  Lack of support | EE | 30-item JSS(20). Job pressure, 10-item subscale (α=.83); lack of support, 10- item subscale (α=.83) | EE assessed via subscale of a 22-item version of the MBI (39) (α= not reported) | Logistic regression: adjusted OR and 95% CI | Lack of support (OR 1.8, 95% CI: 1.5-2.2, p<.001) and job pressure (OR 2.1, 95% CI: 1.8-2.5, p<.001) were significant predictors of EE | Yes: anxiety, depression, DP, PA, subjective health complaints, suicidal ideation, age and gender | High |
| Houdmont *et al.* (8) |  | ≥ 49h/week = long working hours | EE | Respondents asked how many hours they worked in a typical week, excluding overtime, plus the number of hours of overtime. Responses were summed to produce overall weekly working hours (α= not reported) | EE assessed via 9-item subscale of the Human services version of the MBI (MBI-HSS) (36) (α= not reported) | Binary logistic regression:  adjusted OR and 95% CI | 51% (*n*=620) scored high EE. The odds of high EE in the long working hours group were double that of the normal working hours group following adjustment (OR 1.99, 95% CI: 1.52–2.59, p<.05) | Yes: age, gender, rank, departmental tenure and years of police service | High |
| Martinussen *et* *al.* (10) |  | Overtime work  Work conflicts  Leadership  Autonomy  Social support from co-workers and supervisors | EE | Overtime, 1 item (α=not reported);  leadership, 1 item (α= not reported);  work conflict, 2 items (α=.70); autonomy, 2- item subscale, (α=.68); social support from co-workers and supervisors, 8 items (α=.88) | EE 5-item subscale of the 16-item MBI General Survey  (MBI-GS) (36) (α=.86) | Hierarchical multiple regression: standardised beta coefficients (β) | Social support (β=-.25, p<.05) was significantly associated with EE, whereas leadership (β=-.11, p= *ns*), work conflict (β=.01, *ns*), overtime work (β=.07, p= *ns*) or autonomy (β= -.10, p=*ns*) were not | Yes: exposure variables stated^a^, age and gender | High |
| McCarty *et al.* (11) |  | Unfairness of the organisation  Social support from co-workers and supervisors | EE | Unfairness of the organisation, 3-item subscale (α=.75); social support from co-workers and supervisors, 4-item subscale (α=.78) | EE, 4 item subscale (α=.91) from the 22 item MBI (40) | Ordinary least squares regression: unstandardised beta coefficients (b) | Social support (b =-.44, p<.05) and unfairness of the organisation (b=.31, p<.05) were significant predictors of EE | No | Intermediate |
| Mostert *et al.* (14) |  | Job demands  Lack of resources | EE | Police Stress Inventory (41) was used. Previous study (41) demonstrated  job demands (α=.92); lack of resources (α=.92) | EE subscale of the MBI-GS(37) (α=.88) | Multiple regression: standardised beta coefficients (β) | Job demands (β=.22, p<.001) and lack of resources (β=.20, p<.01) as significant predictors of EE | Yes: exposure variables stated^a^, conscientiousness, emotional stability, agreeableness, extraversion, age, gender, race (coloured), race (Indian) | High |
| Xavier *et al*. (15) |  | Administrative/organisational pressure  Police stress | EE | PSS  (α= not reported) | MBI-HSS  (α= not reported) | Correlation= (r) | Police stress (r=.256, p<.01,1-tailed) and administrative/organisational pressure (r=.310, p<.01,1-tailed) were significantly correlated with EE | No | Low |
| Backteman-Erlanson *et al*. (4) |  | Organisational culture  Organisational climate  Leadership  Demand  Decision latitude  Social support at work | DP | Dallner’’s QPSNordic questionnaire(34) which has 118 items, divided among 26 subscales measured organisational culture, 6-item subscale; organisational climate, 5-item subscale; leadership, 8 item subscale. For the three subscales (α=.71-.78)  (35) demand-control questionnaire measured demand, 5- item subscale (α=.78); decision latitude, 6-item subscale (α=0.53); social support at work, 6-item subscale (α=.83) | A Swedish version of the MBI(36-38) assessed DP via 5-item subscale (α=.74) | Univariate logistic regression: univariate adjusted OR and 95% CI by gender | Organisational culture was significantly associated with DP in both female (OR 1.49, 95% CI:1.11-1.99, p<.05) and male police (OR 1.59, 95% CI:1.12- 2.25, p< 0.05). Organisational climate was significantly associated with DP in female police (OR 1.64, 95% CI: 1.22-2.19, p<.05), however not in male police (OR 1.27, 95% CI: 0.94- 1.73, p=*ns*). Social support was significantly associated with DP in both female (OR 1.62, 95% CI: 1.06-2.48, p<.05) and male police (OR 2.18, 95% CI:1.28-3.71, p<.05). Demand demonstrated a significant association with DP in both female (OR 2.54, 95% CI:1.57-4.13, p<.05) and male police (OR 1.96, 95% CI: 1.20-3.20, p<.05). As did decision latitude in both male (OR 2.68, 95% CI: 1.37-5.24, p<.05) and female police (OR 1.77, 95% CI:1.05-2.99, p<.05). Leadership did not demonstrate a significant association with DP in both male (OR 0.85, 95% CI:0.62-1.15, p=*ns*) and female police (OR 0.94, 95% CI: 0.73-1.22, p=*ns*) | Yes: Age | High |
| Berg *et al*. (5) |  | Job pressure  Lack of support | DP | 30-item JSS(20). Job pressure, 10- item subscale (α=.83); lack of support, 10-item subscale (α=.83) | 22-item version of the MBI (39) (α= not reported) | Logistic regression: adjusted OR and 95% CI | Lack of support (OR 0.9, 95% CI: 0.8-1.1, p=*ns*) and job pressure (OR 0.9, 95% CI:0.8-1.1, p=*ns*) were not significantly predictors of DP | Yes: anxiety, depression, PA, EE, subjective health complaints, suicidal ideation, age and gender | High |
| Houdmont *et al.* (8) |  | ≥ 49h/week = long working hours | DP | Respondents asked how many hours they worked in a typical week, excluding overtime, plus the number of hours of overtime. Responses were summed to produce overall weekly working hours (α= not reported) | DP assessed via 5-item subscale of the MBI-HSS (36) (α= not reported) | Binary logistic regression:  adjusted OR and 95% CI | 47% (*n*=573) scored high DP. Long working hours were associated with high DP (OR 1.30, 95% CI: 1.00-1.71, p<.05) | Yes: age, gender, rank, departmental tenure and years of police service | High |
| Martinussen *et* *al.* (10) |  | Overtime work  Work conflicts  Leadership  Autonomy  Social support | DP | Overtime, 1 item (α=not reported);  leadership, 1 item (α= not reported);  work conflict, 2 items (α=.70); autonomy, 2-item subscale, (α=.68); social support from co-workers and supervisors, 8 items (α=.88) | DP assessed via 5-item subscale of the 16-item  MBI-GS(36) (α=.80) | Hierarchical multiple regression: standardised beta coefficients (β) | Leadership was significantly associated with DP  (β=-.24, p<.01), as was social support (β=-.33, p<.001). Whereas work conflict (β=.07, p=*ns*), overtime work (β=.06, p=*ns*), and ‘autonomy’  (β=-.04, p=*ns*) were not | Yes: exposure variables stated^a^, age and gender | High |
| Mostert *et al.* (14) |  | Job demands  Lack of resources | DP | Police Stress Inventory (41) was used. Previous study by Pienaar and Rothmann’s (41) demonstrated  job demands (α=.92); lack of resources (α=.92) | DP subscale of the MBI-GS(37) (α=.79) | Multiple regression: standardised beta coefficients (β) | Job demands (β=.11, p<.001) and lack of resources (β=.17, p=.02) were significant predictors of DP | Yes: exposure variables stated^a^, conscientiousness, emotional stability, agreeableness, extraversion, age, gender, race (coloured) and race (Indian) | High |
| Xavier *et al*. (15) |  | Administrative/organisational pressure  Police stress | DP | PSS (α= not reported) | MBI-HSS  (α= not reported) | Correlation= (r) | Police stress (r=.165, p<.01, 1-tailed) and administrative/organisational pressure (r=.218, p<.01, 1-tailed) were significantly correlated with DP |  | Low |
| Berg *et al*. (5) |  | Job pressure  Lack of support | PA | 30-item JSS(20). Job pressure, 10-item subscale (α=.83); lack of support, 10- item subscale (α=.83) | 22-item version of the MBI (39) (α= not reported) | Logistic regression: adjusted OR and 95% CI | Job pressure was significantly associated with PA (OR 1.3, 95% CI: 1.1-1.6, p<.001), but lack of support was not (OR 1.1, 95% CI: 0.9-1.2, p=*ns*) | Yes: anxiety, depression, EE, DP, subjective health complaints, suicidal ideation, age and gender | High |
| Houdmont *et al.* (8) |  | ≥ 49h/week = long working hours | PA | Respondents asked how many hours they worked in a typical week, excluding overtime, plus the number of hours of overtime. Responses were summed to produce overall weekly working hours (α= not reported) | PA assessed via 8-item subscale of the MBI-HSS (36) (α= not reported) | Binary logistic regression:  adjusted OR and 95% CI | 68% (*n*=838) scored low PA. Long working hours were not significantly associated with PA (OR 0.99, 95% CI: 0.75-1.32, p= *ns*) | Yes: age, gender, rank, departmental tenure and years of police service | High |
| Martinussen *et* *al.* (10) |  | Overtime work  Work conflict  Leadership  Autonomy  Social support | PA | Overtime, 1 item (α=not reported);  leadership, 1 item (α= not reported);  work conflict, 2 items developed by McKeen & Burke (42) (α=.70); autonomy, 2- item subscale, (α=.68); social support from co-workers and supervisors, 8 items used by (43) (α=.88) | PA assessed via 6-item subscale of the  MBI-GS(36) (α=.79) | Hierarchical multiple regression: standardised beta coefficients (β) | Social support was significantly associated with PA (β=.23, p<.01). Whereas leadership (β=.13, p= *ns*), work conflict (β=-.03, p=*ns*), overtime work (β=.01, p=*ns*) and autonomy (β=.09, p=*ns*) were not | Yes: age, gender | High |
| Xavier *et al*. (15) |  | Administrative/organisational pressure  Police stress | PA | PSS (α= not reported) | MBI-HSS  (α= not reported | Correlation= (r) | Police stress (r=.167, p<.01, 1-tailed) and administrative/organisational pressure (r=.152, p<.01, 1- tailed) were significantly correlated with PA | No | Low |
| Berg *et al*. (5) |  | Job pressure  Lack of support | Suicidal ideation | 30-item JSS(20). Job pressure, 10-item subscale (α=.83); lack of support, 10- item subscale (α=.83) | Modified version of Paykel *et al*’s.(44) Suicidal Feelings in the General Population questionnaire (α=not reported) | Logistic regression: adjusted OR and 95% CI | Job pressure (OR 0.8, 95% CI: 0.6-1.19, p=*ns*) and lack of support (OR 1.3, 95% CI: 0.9-1.7, p=*ns*) were not significantly associated with suicidal ideation | Yes: anxiety, depression, DP, PA, EE, subjective health complaints, age and gender | High |

References

1. Adams GA, Buck J. Social stressors and strain among police officers: It’s not just the bad guys. Criminal Justice and Behavior. 2010;37(9):1030-40.

2. Adebayo DO, Sunmola AM, Udegbe IB. Workplace fairness and emotional exhaustion in Nigeria police: The moderating role of gender. Anxiety, Stress & Coping: An International Journal. 2008;21(4):405-16.

3. Arial M, Gonik V, Wild P, Danuser B. Association of work related chronic stressors and psychiatric symptoms in a Swiss sample of police officers; a cross sectional questionnaire study. International Archives Of Occupational And Environmental Health. 2010;83(3):323-31.

4. Backteman-Erlanson S, Padyab M, Brulin C. Prevalence of burnout and associations with psychosocial work environment, physical strain, and stress of conscience among Swedish female and male police personnel. Police Practice & Research: An International Journal. 2013;14(6):491-505.

5. Berg A, Hem E, Lau B, Ekeberg Ø. An exploration of job stress and health in the Norwegian police service: a cross sectional study. Journal Of Occupational Medicine And Toxicology 2006;1:26-.

6. Chen H, Chou Fh, Chen M, Su S, Wang S, Feng W, et al. A survey of quality of life and depression for police officers in Kaohsiung, Taiwan. Quality of Life Research: An International Journal of Quality of Life Aspects of Treatment, Care & Rehabilitation. 2006;15(5):925-32.

7. Crank JP, Regoli R, Hewitt JD, Culbertson RG. Institutional and organizational antecedents of role stress, work alienation, and anomie among police executives. Criminal Justice and Behavior. 1995;22(2):152-71.

8. Houdmont J, Randall R. Working hours and common mental disorders in English police officers. Occupational medicine (Oxford, England). 2016.

9. Janzen BL, Muhajarine N, Zhu T, Kelly IW. Effort-reward imbalance, overcommitment, and psychological distress in Canadian police officers. Psychological Reports. 2007;100(2):525-30.

10. Martinussen M, Richardsen AM, Burke RJ. Job demands, job resources, and burnout among police officers. Journal of Criminal Justice. 2007;35(3):239-49.

11. McCarty WP, Skogan WG. Job-related burnout among civilian and sworn police personnel. Police Quarterly. 2013;16(1):66-84.

12. Morash M, Haarr R, Hoon Kwak D. Multilevel influences on police stress. Journal of Contemporary Criminal Justice. 2006;22(1).

13. Morash M, Kwak D, Hoffman V, Lee C, Cho S, Moon B. Stressors, coping resources and strategies, and police stress in South Korea. Journal of Criminal Justice. 2008;36(3):231-9.

14. Mostert K, Rothmann S. Work-related well-being in the South African Police Service. Journal of Criminal Justice. 2006;34(5):479-91.

15. Xavier P, Prabhakar K. A study of police stress and burnout among Tamil Nadu police. International Journal of Pharmaceutical Sciences Review and Research. 2016;38(2):159-61.

16. Cullen F, Link B, Wolf N, Frank J. The social dimensions of police officer stress. Justice Quarterly. 1989;2:507-33.

17. Morash M, Haarr RN. Gender, workplace problems, and stress in policing. Justice Quarterly. 1995;12(1):113-40.

18. Cullen FT, Lemming T, Link BG, Wozniak JF. The impact of social supports on police stress. Criminology. 1985;3:503-22.

19. Haar RN, Morash M. Gender, race, and strategies of coping with occupational stress in policing. Justice Quarterly. 1999;16:303-36.

20. Spielberger CD, Vagg PR. Professional manual for the Job Stress Survey (JSS). Research edition. Odessa, FL: Psychological Assessment Resources; 1999.

21. Zigmond AS, Snaith RP. The Hospital and Anxiety Depression scale. Acta Psychiatrica Scandinavica. 1983;67:361-70.

22. Chou FH, Su TT, Ou-Yang WC, Chien IC, Lu MK, Chou P. Establishment of a disaster related psychological screening test. Australian & New Zealand Journal of Psychiatry. 2003;37(1):97-103.

23. Cortina L, Magley V, Williams J, Langhout R. Incivility in the workplace: incidence and impact. Journal of Occupational Health Psychology. 2001;6:64-80.

24. Caplan RD, Cobb S, French FRP, Van Harrison R, Pinneau SR. Job demands and worker health. Michigan: University of Michigan, Institute for Social Research; 1980.

25. Langner TS. A twenty-two item screening score of psychaitric symptom indicating impairment. Journal of Health and Human Behaviour. 1962;2(269-276).

26. Amiel R. La notion de santé mentale et son évaluation dans les études épidémiologiques à visées préventives en médecine du tra- vail et en santé communautaire. Archives des Maladies Professionnelles et de l’Environment. 1986;47:1-14.

27. Johnson DR, Meile RL. Does dimensionality bias in Langner’s 22-item index affect the validity of social status comparisons? An empirical investigation. Journal of Health and Social Behavior. 1981;22:415-33.

28. Goldberg D, Williams P. Users guide to the General Health Questionnaire. Windsor, UK: NFER-Nelson; 1988.

29. Siegrist J, Starke D, Chandola T, Godin I, Marmot M, Niedhammer L, et al. The measurement of effort-reward imbalance at work: European comparisons. Social Science & Medicine. 2004;58(1483-1499).

30. Kessler RC, Andrews G, Colpe L, Hiripi R, Mroczek D, Normand S, et al. Short screening scales to monitor population prevalences and trends in nonspecific distress. Psychological medicine. 2002;32:959-76.

31. Wharton AS. The affective consequences of service work. Work and Occupations. 1993;20(205-232).

32. Van Yperen NW. Informational support, equity and burnout: the moderating effect of self-efficacy. Journal of Occupational and Organizational Psychology. 1998;71(29-33).

33. Maslach C, Jackson S. The Maslach burnout inventory. Palo Alto,CA: Consunting Psychologists; 1986.

34. Dallner M. Validation of the general nordic questionnaire (QPSNordic) for psychological and social factors at work. Solna: Nordic Council of Ministers; 2000.

35. Karasek RA, Theorell T. Healthy work, stress, productivity and the reconstructon of working life. New York: Basic Books; 1990.

36. Maslach C, Jackson SE, Leiter MP. The Maslach burnout inventory. 3rd ed. Palo Alto, CA: Consulting Psychologists; 1996.

37. Maslach C, Jackson SE, Leiter MP, Schaufeli WB, Schwab RL. Maslach burnout inventory (MBI): manual. Palo Alto, CA: Consulting Psychologists; 1981.

38. Hallsten L. Utbrand i jobbet in Swedish burnout at work Stockholm: National Institute for Working Life; 1985.

39. Maslach C, Schaufeli WB, Leiter MP. Job burnout. Annual Review of Psychology. 2001;52(397-422).

40. Cooper CL, Dewe PJ, O’’Driscoll MP. A special form of strain: job realted burnout. Organisational stress: a review and critique of theory, research and applications. London: Sage; 2001. p. 79-116.

41. Pienaar J, Rothmann S. Job stress in the South African police services. Fifteenth conference of the South African Institute for Management Scientists; Potchefstroom, South Africa2003.

42. McKeen CA, Burke RJ. Work experiences and career success of a professional women: study design and preliminary findings. Canadian Journal of Administrative Sciences. 1991;8:251-8.

43. Himle DP, Jayaratne SD, Thyness P. Buffering effects on four social support types on burnout among social workers. Social Work Research and Abstracts. 1991;27:22-7.

44. Paykel ES, Myers JK, Lindenthal J, Tanner J. Suicidal feelings in the general population: a prevalence study. British Journal of Psychiatry. 1974;124:460-9.
