# Supplementary material for: The relationship between organisational stressors and mental wellbeing within police officers: a systematic review
Source: BMC Public Health. 2019 Oct 15;19:1286. doi: 10.1186/s12889-019-7609-0 (PMC6792329; doi:10.1186/s12889-019-7609-0)
Supplement: Supplementary file 6 — Additional file 6: Tables S12 and S13. Process Adopted to Determine Overall Magnitude of Association of Included Studies by Outcome (Table S12) and Overall Degree of Evidence Grade by MW Outcome (Table S13). [file 12889_2019_7609_MOESM6_ESM.docx]

The Relationship between Organisational Stressors and Mental Wellbeing within Police Officers: A Systematic Review

Additional File 6

File Format: DOC

Title: Table S12 and S13

Description: Process Adopted to Determine Overall Magnitude of Association of Included Studies by Outcome (Table S12) and

Overall Degree of Evidence Grade by MW Outcome (Table S13)

Table S12

Process Adopted to Determine Overall Magnitude of Association of Included Studies by Outcome

| Mental Wellbeing Outcome (s) | Study ID(s) | Individual effect measures grades. and corresponding score (points) | High (3 pts) | Intermediate  (2 pts) | | | Low  (1 pt) | No association  (0 pts) | | | Unclear  (0 pts) | Overall magnitude of association* | Overall magnitude of association: RAG threshold** |
| --- | --- | --- | --- | --- | --- | --- | --- | --- | --- | --- | --- | --- | --- |
| Occupational Stress | Crank *et al*. (1) Morash *et al*. (2) Morash *et al.* (3) | Number of effect sizes of the following grades within included studies | 0 | 1 | | | 7 | 5 | | | 0 | 9/3 = 3.0 | Intermediate (++) |
|  |  | Score applied to effect measures in included studies | - | 2*1 | | | 7*1 | - | | | - |  |  |
| Anxiety | Berg *et al*. (4) | Number of effect sizes of the following grades within included studies | 0 | 1 | | | 0 | 1 | | | 0 | 2/1= 2.0 | Intermediate (++) |
|  |  | Score applied to effect measures in included studies | - | 1*2 | | | - | - | | | - |  |  |
| Depression | Berg *et al*. (4)  Chen *et al*. (5) | Number of effect sizes of the following grades within included studies | 0 | 2 | | | 0 | 2 | | | 0 | 4/2= 2.0 | Intermediate (++) |
|  |  | Score applied to effect measures in included studies | - | 2*2 | | | - | - | | | - |  |  |
| Psychiatric Symptoms (PS) or Psychological Distress (PD) | Adams *et al*. (6) Arial *et al*. (7)  Houdmont *et al.* (8)  Janzen *et al*. (9) | Number of effect sizes of the following grades within included studies | 1 | | 3 | 3 | | | 0 | 0 | | 12/3= 4.0 | High (++) |
|  |  | Score applied to effect measures in included studies | 1*3 | | 3*2 | 3*1 | | | - | - | |  |  |
| Burnout | Xavier *et al.* (10) | Number of effect sizes of the following grades within included studies | 0 | 0 | | | 2 | 0 | | | 0 | 2/1= 2.0 | Intermediate (++) |
|  |  | Score applied to effect measures in included studies | - | - | | | 2*1 | - | | | - |  |  |
| Emotional Exhaustion (EE) | Adams *et al*. (6)  Adebayo *et al*. (11)  Berg *et al*. (4)  Backteman-Erlanson *et al*. (12)  Houdmont *et al.* (8)  Martinussen *et* *al.* (13)  Mostert *et al.* (14)  McCarty *et al.* (15)  Xavier *et al.* (10) | Number of effect sizes of the following grades within included studies | 1 | 7 | | | 9 | 5 | | | 0 | 26/4=6.5 | High (+++) |
|  |  | Score applied to effect measures in included studies | 1*3 | 7*2 | | | 9*1 | - | | | - |  |  |
| Depersonalisation (DP) | Berg *et al*. (4)  Backteman-Erlanson *et al*. (12)  Houdmont *et al.* (8)  Martinussen *et* *al.* (13)  Mostert *et al.* (14)  Xavier *et al.* (10) | Number of effect sizes of the following grades within included studies | 0 | 5 | | | 7 | 6 | | | 0 | 17/3=5.7 | High (+++) |
|  |  | Score applied to effect measures in included studies | - | 5*2 | | | 7*1 | - | | | - |  |  |
| Personal Accomplishment (PA) | Berg *et al*. (4) Houdmont *et al.* (8)  Martinussen *et* *al.* (13)  Xavier *et al.* (10) | Number of effect sizes of the following grades within included studies | 0 | 0 | | | 4 | 6 | | | 0 | 4/2= 2.0 | Intermediate (++) |
|  |  | Score applied to effect measures in included studies | - | - | | | 4*1 | - | | | - |  |  |
| Suicidal Ideation | Berg *et al*. (4) | Number of effect sizes of the following grades within included studies | 0 | 0 | | | 0 | 2 | | | 0 | 0 | Low (+) |
|  |  | Score applied to effect measures in included studies | - | - | | | - | - | | | - |  |  |

*Note*. Individual grades were given to the effect size of each stressor and outcome relationship within each study (high, intermediate, low, no association, unclear). These studies were then graded using a weighted average (WA) scale, wherein a High association (+++ = *3 points*), an Intermediate association (++ = *2 points*), and a Low association (+ = *1 point*). For No association (-) and unclear (±) a *0 points scale* was allocated. In calculating the overall mean score, a weighted average was calculated for each outcome (sum of scores applied to effect measures for MW outcome/ no of grades applied to each MW outcome). **The overall magnitude of association of included studies by outcome was then graded accordingly using the RAG threshold: high (+++); ≥4; intermediate (++), 2.0- 3.9; low/ no association (+) 0-1.9.

Table S13

Overall Degree of Evidence Grade by MW Outcome

| Mental Wellbeing Outcome(s) | Study ID | NOS grade | Adjustment by confounder(s) results | Overall degree of evidence* |
| --- | --- | --- | --- | --- |
| Occupational Stress | Crank *et al*. (1) | High | No adjustment | Strong |
|  | Morash *et al.* (3) | High | Full adjustment |  |
|  | Morash *et al*. (2) | High | Full adjustment |  |
| Anxiety | Berg *et al*. (4) | High | Full adjustment | Insufficient |
| Depression | Berg *et al*. (4) | High | Full adjustment | Insufficient |
|  | Chen *et al*. (5) | High | Partial adjustment |  |
| Psychiatric Symptoms (PS) or Psychological Distress (PD) | Adams *et al*. (6) | High | Partial adjustment | Strong |
|  | Arial *et al*. (7) | High | Full adjustment |  |
|  | Houdmont *et al.* (8) | High | Partial adjustment |  |
|  | Janzen *et al*. (9) | High | Partial adjustment |  |
| Burnout | Xavier *et al.* (10) | Low | No adjustment | Insufficient |
| Emotional Exhaustion (EE) | Adams *et al*. (6) | High | Partial adjustment | Strong |
|  | Adebayo *et al*. (11) | High | Partial adjustment |  |
|  | Backteman-Erlanson *et al*. (12) | High | Partial adjustment |  |
|  | Berg *et al*. (4) | High | Full adjustment |  |
|  | Houdmont *et al.* (8) | High | Partial adjustment |  |
|  | Martinussen *et* *al.* (13) | High | Full adjustment |  |
|  | McCarty *et al..* (15) | Intermediate | No adjustment |  |
|  | Mostert *et al.* (14) | High | No adjustment |  |
|  | Xavier *et al.* (10) | Low | No adjustment |  |
| Depersonalisation (DP) | Backteman-Erlanson *et al*. (12) | High | Partial adjustment | Strong |
|  | Berg *et al*. (4) | High | Full adjustment |  |
|  | Houdmont *et al.* (8) | High | Partial adjustment |  |
|  | Martinussen *et* *al.* (13) | High | Full adjustment |  |
|  | Mostert *et al.* (14) | High | Full adjustment |  |
|  | Xavier *et al.* (10) | Low | No adjustment |  |
| Personal Accomplishment (PA) | Berg *et al*. (4) | High | Full adjustment | Strong |
|  | Houdmont *et al.* (8) | High | Partial adjustment |  |
|  | Martinussen *et* *al.* (13) | High | Full adjustment |  |
|  | Xavier *et al.* (10) | Low | No adjustment |  |
| Suicidal Ideation | Berg *et al*. (4) | High | Full adjustment | Insufficient |

*Note.* Degree of evidence of included studies by outcome classified as strong, moderate or insufficient. Strong evidence (+++): *Consistent findings in more than 2 studies of high quality. At least one study has adjusted for participant demographics AND additional exposure variables*.; moderate evidence (++): *Consistent findings in 2 studies of high quality or one high quality study and one intermediate quality study, or between more than 2 studies of intermediate quality. At least one study has adjusted for participant demographics OR additional exposure variables*; insufficient evidence *(+): Identification of only one study or inconsistent findings across studies*.

References

1. Crank JP, Regoli R, Hewitt JD, Culbertson RG. Institutional and organizational antecedents of role stress, work alienation, and anomie among police executives. Criminal Justice and Behavior. 1995;22(2):152-71.

2. Morash M, Kwak D, Hoffman V, Lee C, Cho S, Moon B. Stressors, coping resources and strategies, and police stress in South Korea. Journal of Criminal Justice. 2008;36(3):231-9.

3. Morash M, Haarr R, Hoon Kwak D. Multilevel influences on police stress. Journal of Contemporary Criminal Justice. 2006;22(1).

4. Berg A, Hem E, Lau B, Ekeberg Ø. An exploration of job stress and health in the Norwegian police service: a cross sectional study. Journal Of Occupational Medicine And Toxicology 2006;1:26-.

5. Chen H, Chou Fh, Chen M, Su S, Wang S, Feng W, et al. A survey of quality of life and depression for police officers in Kaohsiung, Taiwan. Quality of Life Research: An International Journal of Quality of Life Aspects of Treatment, Care & Rehabilitation. 2006;15(5):925-32.

6. Adams GA, Buck J. Social stressors and strain among police officers: It’s not just the bad guys. Criminal Justice and Behavior. 2010;37(9):1030-40.

7. Arial M, Gonik V, Wild P, Danuser B. Association of work related chronic stressors and psychiatric symptoms in a Swiss sample of police officers; a cross sectional questionnaire study. International Archives Of Occupational And Environmental Health. 2010;83(3):323-31.

8. Houdmont J, Randall R. Working hours and common mental disorders in English police officers. Occupational medicine (Oxford, England). 2016.

9. Janzen BL, Muhajarine N, Zhu T, Kelly IW. Effort-reward imbalance, overcommitment, and psychological distress in Canadian police officers. Psychological Reports. 2007;100(2):525-30.

10. Xavier P, Prabhakar K. A study of police stress and burnout among Tamil Nadu police. International Journal of Pharmaceutical Sciences Review and Research. 2016;38(2):159-61.

11. Adebayo DO, Sunmola AM, Udegbe IB. Workplace fairness and emotional exhaustion in Nigeria police: The moderating role of gender. Anxiety, Stress & Coping: An International Journal. 2008;21(4):405-16.

12. Backteman-Erlanson S, Padyab M, Brulin C. Prevalence of burnout and associations with psychosocial work environment, physical strain, and stress of conscience among Swedish female and male police personnel. Police Practice & Research: An International Journal. 2013;14(6):491-505.

13. Martinussen M, Richardsen AM, Burke RJ. Job demands, job resources, and burnout among police officers. Journal of Criminal Justice. 2007;35(3):239-49.

14. Mostert K, Rothmann S. Work-related well-being in the South African Police Service. Journal of Criminal Justice. 2006;34(5):479-91.

15. McCarty WP, Skogan WG. Job-related burnout among civilian and sworn police personnel. Police Quarterly. 2013;16(1):66-84.
