# Supplementary material for: The relationship between organisational stressors and mental wellbeing within police officers: a systematic review
Source: BMC Public Health. 2019 Oct 15;19:1286. doi: 10.1186/s12889-019-7609-0 (PMC6792329; doi:10.1186/s12889-019-7609-0)
Supplement: Supplementary file 7 — Additional file 7: Table S14. Eligible Studies following Title and Abstract Screening, which could not be accessed in Full-text (Table S14). [file 12889_2019_7609_MOESM7_ESM.docx]

The Relationship between Organisational Stressors and Mental Wellbeing within Police Officers: A Systematic Review

Additional File 7

File Format: DOC

Title: Table S14

Description: Eligible Studies following Title and Abstract Screening, which could not be accessed in Full-text (Table S14)

Table S14

Eligible Studies following Title and Abstract Screening, which could not be accessed in Full-text

| Bibliographic Citation | Study Type | Attempt to Access Study |
| --- | --- | --- |
| Abdollahi MK. *The effects of organizational stress on line staff law enforcement officers*. US: ProQuest Information & Learning; 2004. | Thesis | AC  RG  SA |
| Aksoy M, Dolu O, Gul SK. *Police stress 16/11/2005 annual meeting*. Toronto: American Society of Criminology;2005. | Conference paper | N |
| Amininejad A. *The stressors associated with police work: considerations for mental health consultation*. US: ProQuest Information & Learning; 2012. | Thesis | AC  SA |
| Anderson EM. *Stress and its correlates: an empirical investigation among North Dakota peace officers*. US: ProQuest Information & Learning; 1996. | Thesis | AC  SA |
| Banerjee U, Gupta HN. Moderating effect of social support in occupational stress - strain relationship. *Journal of the Indian Academy of Applied Psychology*. 1996;22(1-2):27-34. | Journal article | AC |
| Bezerra CM, Minayo MCS, Constantino P. Occupational stress among female police officers. *Ciencia e Saude Coletiva.* 2013;18(3):657-66. | Journal article | AC  SA |
| Boyden RD. *The impact of career expectation stress and job perception stress on morale and job performance of police officers employed in agencies with 100 or fewer uniformed personnel.* US: ProQuest Information & Learning; 2011. | Thesis | AC  SA |
| Brough P. Comparing the influence of traumatic and organizational stressors on the psychological health of police, fire, and ambulance officers. *International Journal of Stress Management.* 2004;11(3):227-44. | Journal article | AC |
| Buker H. *A comparative examination of organizational climate, job satisfaction and workplace stress: The case of turkish national police 16/11/2005 annual meeting*. Toronto: American Society of Criminology;2005. | Conference paper/unpublished manuscript | N  SA |
| Burke RJ, Mikkelsen A. Burnout among Norwegian police officers: Potential antecedents and consequences. *International Journal of Stress Management.* 2006;13(1):64-83. | Journal article | RG  SA  AC |
| Donovan JG. *Stressful experiences of urbanized small town police officers: a case study*. US: ProQuest Information & Learning; 2014. | Thesis | AC  SA |
| Gharibian E. *Associations of job strain, health-related quality of life, mental health stigma and seeking mental health treatment among police officers.* US: ProQuest Information & Learning; 2016. | Journal article | AC |
| Ghosh S, Chaudhury PK, Zaman RU. A study of stress, quality of life and psychiatric morbidities in police personnel. Indian J Psychiatry. 2011; 53(5 SUPPL. 1):S73. | Journal article | N  SA |
| Gray LK. *Emergency service providers' organizational climate and its role in the development of traumatic stress and posttraumatic growth*. US: ProQuest Information & Learning; 2009. | Thesis | N  SA |
| Hedgley CL. *Perceived organizational stress of law enforcement officers and their police organization as measured by the Pressure Management Indicator (PMI).* US: ProQuest Information & Learning; 2008. | Thesis | N  SA |
| Heist KB. Object relations and occupational stress: An examination of police officers and vulnerability to stress. US: ProQuest Information & Learning; 2014. | Thesis | N  SA |
| Kaya M. *The effects of perceived organizational justice on police job satisfaction, job involvement and job stress: A case of a turkish national police.* US: ProQuest Information & Learning; 2015. | Thesis | SA |
| Kirkcaldy B. Job stress and satisfaction: international police officers. *Psychol Rep*. 1993;72(2):386. | Journal article | RG  AC  SA |
| Kirkcaldy BD, Furnham AF. Coping, seeking social support and stress among German police officers. *European Review of Applied Psychology / Revue Européenne de Psychologie Appliquée. 1995*;45(2):121-6. | Journal article | N  SA |
| Lambert BE. *Texas community college police officer stress: an empirical investigation*. US: ProQuest Information & Learning; 1997. | Thesis | N  SA |
| Laufersweiler DL. *Organizational stress: A tri-level analysis of the factors which affect perceived stress*. US: ProQuest Information & Learning; 1995. | Thesis | N  SA |
| Lima C, Maia A, Ferreira R, Magalhaes A, Nunes H, Pinheiro S, *et al*. PTSD and quality of life among fire-fighters and municipal police forces*. European Psychiatry*. 2016; 33: S513. | Journal article | RG  SA  AC |
| Mendelson G. Occupational stress part 1: An overview. *Journal of Occupational Health and Safety - Australia and New Zealand.* 1990;6(3):175-80. | Journal article | N  SA |
| Mendelson G. Occupational stress part 2: Occupations at risk. *Journal of Occupational Health and Safety - Australia and New Zealand.* 1990;6(3):181-8. | Journal article | N  SA |
| Mendelson G. Occupational stress part 3: Medico-legal aspects. *Journal of Occupational Health and Safety - Australia and New Zealand.* 1990;6(3):189-97. | Journal article | N  SA |
| Moreno BR. *Organizational stress: The unseen enemy of police.* US: ProQuest Information & Learning; 2011. | Thesis | AC |
| Morton T. *Job-related stressors on African-American police officers.* US: ProQuest Information & Learning; 2004. | Thesis | N  SA |
| Page KS, Jacobs SC. Surviving the shift: Rural police stress and counselling services. *Psychol Serv*. 2011;8(1):12-22. | Journal article | AC |
| Parsekar SS, Singh MM, Bhumika TV. Occupation-related psychological distress among police constables of Udupi taluk, Karnataka: A cross-sectional study. *Indian J Occup Environ Med*. 2015;19(2):80-3. | Journal article | AC |
| Pinto LW, Figueiredo AEB, de Souza ER. Psychic suffering among civil police officers in Rio de Janeiro State*. Ciencia e Saude Coletiva*. 2013;18(3):633-44. | Journal article | AC* |
| Rodichok GJ. *A quantitative and qualitative survey of job stress among African-American police officers*. US: ProQuest Information & Learning; 1995. | Thesis | N  SA |
| Setti I, Argentero P. The influence of operational and organizational stressors on the well-being of municipal police officers. *La Medicina Del Lavoro*. 2013;104(5):368-79. | Journal article | RG  AC |
| Spina E. *The perception of stress with law enforcement personnel.* US: ProQuest Information & Learning; 2006. | Thesis | N  SA |
| Thomas-Riddle FR. *The relationship between life stress, work stress, and traumatic stress and Burnout and cynicism in police officers*. US: ProQuest Information & Learning; 2000. | Thesis | N  SA |
| Violanti JM. Introduction to special issue: stress and health in law enforcement. *Int J Emerg Ment Health*. 2011;13(4):209-10. | Journal article | RG  AC |
| Violanti JM, Aron F. Sources of police stressors, job attitudes, and Psychological Distress. *Psychol Rep.* 1993;72(3 Pt 1):899-904. | Journal article | RG  AC |
| Violanti JM, Aron F. Ranking police stressors. *Psychol Rep.* 1994;75(2):824-6. | Journal article | RG  AC |
| Vucovich S. *Posttraumatic stress disorder symptomatology among law enforcement specialties*. US: ProQuest Information & Learning; 2013. | Thesis | N  SA |
| Webb CJ. *A quantitative study of stress factors of supervisory and line-officers within law enforcement.* US: ProQuest Information & Learning; 2010. | Thesis | N  SA |

*Note.* Study author(s)/ thesis student supervisor contacted: AC; Study author(s) contacted to access full-text English language version of study: AC; Research Gate request submitted: RG; Supervisor attempt to access via institutional and journal subscription options: SA; No available contact details for study author(s): N

*exposure variables*; insufficient evidence *(+): Identification of only one study or inconsistent findings across studies*.
